# TBG096 Ameliorates Memory Deficiency in AD Mouse Model via Promoting Neurogenesis and Regulation of Hsc70/HK2/PKM2/LAMP2A Signaling Pathway

**DOI:** 10.3390/ijms26062804

**Published:** 2025-03-20

**Authors:** Danni Chen, Opeyemi B. Fasina, Jiahui Lin, Jiayuan Zeng, Majid Manzoor, Hiroshi Ohno, Lan Xiang, Jianhua Qi

**Affiliations:** 1 College of Pharmaceutical Sciences, Zhejiang University, Yu Hang Tang Road 866, Hangzhou 310058, China; 12019025@zju.edu.cn (D.C.); 11919056@zju.edu.cn (O.B.F.); 22219029@zju.edu.cn (J.L.); 22219069@zju.edu.cn (J.Z.); 11719054@zju.edu.cn (M.M.); 2 RIKEN Center for Integrative Medical Sciences, 1-7-22 Suehirocho, Tsutumiku, Yokohama 230-0045, Japan; hiroshi.ohno@riken.jp

**Keywords:** Alzheimer’s disease, neurogenesis, nerve growth factor, heat-shock cognate protein 70

## Abstract

In previous studies, we isolated a series of novel gentisides with nerve growth factor (NGF)-mimic activities from *Gentiana rigescens* Franch and conducted continuous structure–activity relationship (SAR) studies. Recently, a lead compound named TBG096 was discovered with significant NGF-mimic activity, low toxicity, and ability to pass through the blood–brain barrier (BBB). At the cell level, TBG096 exerts NGF-mimic activity by regulation of heat-shock cognate protein 70 (Hsc70) and downstream proteins. Subsequently, high-fat diet (HFD)-induced Alzheimer disease (AD) mouse models were used to evaluate the anti-AD efficacy of the compound. TBG096 significantly improved the memory dysfunction of AD mice at doses of 0.1, 5, and 20 mg/kg, respectively. In order to elucidate the mechanism of action of the compound against AD, the RNA-sequence analysis of transcriptomics, quantitative real-time polymerase chain reaction (qRT-PCR), immunofluorescence staining, and Western blot analysis were performed using animal samples. TBG096 significantly increased the expression of the Wnt gene family (*Wnt10b*, *Wnt5a*, and *Wnt1*) and the number of mature neurons and newborn neurons in the hippocampus and cerebral cortex of AD mice, respectively. At the same time, it reduced the activity of microglia, astrocyte cells, and expression of inducible nitric oxide synthase (INOS) in the brain. Moreover, this compound significantly increased phosphorylated-adenosine 5′-monophosphate-activated protein kinase (AMPK), Hsc70, and lysosomal-associated membrane protein 2a (LAMP2A) and decreased the expression of hexokinase 2 (HK2), pyruvate kinase M2 (PKM2), amyloid precursor protein (APP), microtubule-associated protein tau (Tau), phosphoryl-Tau, and β-amyloid (Aβ) at the protein level. These results suggest that TBG096 produced the NGF-mimic activity and the anti-AD effect via promoting neurogenesis and modification of the Hsc70/HK2/PKM2/LAMP2A signaling pathway, proposing a potential novel approach to counteracting cognitive decline by developing small molecules that promote neurogenesis and the Hsc70 signaling pathway.

## 1. Introduction

Alzheimer disease (AD) is the most prevalent neurodegenerative condition among the elderly. In comparison to diseases such as cancer, heart disease, and stroke, it has become one of the leading causes of death in individuals over 65 years of age [1]. It is widely accepted that AD arises from a combination of pathological processes, including β-amyloid (Aβ) abnormality, microtubule-associated protein tau (Tau) hyperphosphorylation, neuroinflammation, neurotransmitter imbalance, and neuronal damage [2]. The upstream regulators of these pathological features have not been fully clarified. Since the most widely adopted treatments aimed at reducing the burden of Aβ have not proven effective, it is urgent to find a new treatment for AD. The ideal therapeutic strategy should not only eliminate the pathological features of the disease but also facilitate some functional recovery [3]. Emerging evidence has shown that autophagy system disorders are the key pathological features of AD [4]. Therefore, one of the new strategies is to find small molecules that can promote neurogenesis and regulate autophagy.

Nerve growth factor (NGF) is the first recognized neurotrophic factor and is considered a potential candidate drug for AD treatment [5]. Preclinical evidence highlights the critical role of NGF in neuroprotection, resilience, and cognitive function in models of neuronal injury and aging [6,7]. The PC12 cell bioassay system has been established as a reliable method for investigating potential drugs with NGF-mimic activity. Following NGF treatment, PC12 cells cease dividing and start to differentiate, similar to neurons. However, NGF faces challenges in crossing the blood–brain barrier (BBB) due to its high polarity and large molecular weight, which limits its therapeutic potential. Consequently, developing small molecules with NGF-mimic activity is a promising approach for AD treatment.

Adult neurogenesis has been found in some areas of the brain, especially in the hippocampus, during the life of various mammals [8]. Blocking the adult hippocampal neurogenesis aggravates cognitive impairment in the AD mouse model. However, targeting neurogenesis may improve cognitive dysfunction in AD [9]. Wnt signaling plays a major role in regulating adult hippocampal neurogenesis [10], whereas the phosphatidylinositol 3-kinase (PI3K) pathway is implicated in neuroregeneration [11]. Discovering small molecules that can regulate these two signaling pathways and promote neurogenesis is a new idea for the treatment of AD.

Additionally, autophagy plays a crucial regulatory role in neurodegenerative diseases. Mutations in genes associated with autophagy and lysosomal function contribute to the development of various neurodegenerative diseases [12]. Notably, abnormalities in the lysosome and autophagy system within neurons represent an early prominent feature of AD [13,14]. Chaperone-mediated autophagy (CMA) is a lysosomal-dependent selective degradation pathway involved in the pathogenesis of cancer and neurodegenerative diseases. A remarkable aspect of CMA is its ability to selectively degrade proteins based on a recognition motif present in their amino acid sequence [15]. This specificity enables the elimination of particular proteins (substrates that contain the Lys-Phe-Glu-Arg-Gln (KFERQ) motif) without affecting adjacent proteins [16]. One of the key proteins in CMA is heat-shock cognate protein 70 (Hsc70), a molecular chaperone from the family of heat-shock proteins (HSP) [17]. It has been reported that Hsc70 has diverse intracellular functions, including binding to aggregated and misfolded peptides as well as facilitating the degradation of target proteins such as amyloid precursor protein (APP), hexokinase 2 (HK2), and pyruvate kinase M2 (PKM2) through the ubiquitin-proteasome system (UPS) and CMA [18,19,20]. Meanwhile, Hsc70 may help prevent the accumulation of phosphorylated Tau (p-Tau) protein [21]. Consequently, identifying compounds that activate the CMA pathway of Hsc70 to clear misfolded proteins is anticipated to be a promising strategy for preventing or treating AD.

To investigate the activity and mechanisms of compounds for treating AD, selecting an appropriate animal model is critical. Commonly used AD models include aging animals, transgenic animals (such as APP/PS1 double transgenic mice, 3×Tg mice, and 5×FAD mice), and exogenously induced (such as Aβ, D-galactose, and high-fat diet (HFD)-induced AD mice) models. Among these, the HFD-induced AD mouse model is less invasive, effectively simulates AD symptoms, and accelerates pathology under natural conditions. The metabolic defects caused by HFD contribute to AD risk through mechanisms involving neuroinflammation and amyloidosis [22] and usually lead to gut microbiota disorder. For this study, HFD-induced AD mice were utilized.

Previous studies identified several neuritogenic compounds, termed gentisides, from the traditional Chinese medicine *Gentiana rigescens* Franch in PC12 cells. Structure–activity relationship (SAR) analyses of these gentisides led to the synthesis of hundreds of alkyl benzoate derivatives [23]. Activity-based protein profiling (ABPP) identified Hsc70 as the target protein for one of the leading compounds [24]. However, the poor water solubility and low bioavailability of the leading compound necessitated the development of more derivatives with improved properties. From these derivatives, a new lead compound, TBG096, was identified. This report demonstrates that TBG096 exerts NGF-mimic activity and ameliorates memory dysfunction in AD mouse models by promoting neurogenesis and regulation of the Hsc70/HK2/PKM2/LAMP2A signaling pathway.

## 2. Results

### 2.1. SAR of Gentiside Derivatives and Discovery of the Leading Compound with NGF-Mimic Activity

**Figure 1:**
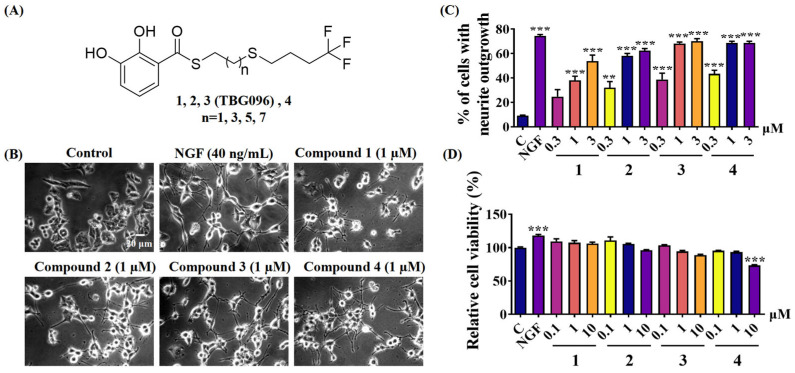
Discovery of the lead compound TBG096. (**A**) Chemical structures of gentisides derivatives **1**–**4**. (**B**) Morphological changes of neurite outgrowth in PC12 cells treated with NGF and compounds **1**–**4**. (**C**) Percentages of PC12 cells with neurite outgrowth after treatment with compounds **1**–**4** at doses of 0.3, 1, and 3 µM for 48 h. (**D**) Relative cell viability in PC12 cells after treatment with **1**–**4** at doses of 0.1, 1, and 10 µM. The data are presented as mean ± SEM, and the experiment was performed in triplicate. ** and *** indicate significant differences in comparison with the control group at *p* < 0.01 and *p* < 0.001, respectively.

Previous studies discovered gentisides with NGF-mimic effects derived from *Gentiana rigescens* Franch, along with the synthesis of hundreds of alkyl benzoate derivatives. These derivatives were designed to enhance NGF-mimic activity while reducing cytotoxicity through the transformation of thioester and the introduction of sulfur atoms in the alkyl chain [23]. However, the water solubility and bioavailability of these derivatives remained insufficient. To address these issues, structural modifications were implemented, including adjustments to the alkyl chain length to improve water solubility and substitution of the terminal methyl group’s hydrogen atom with fluorine to enhance bioavailability. The synthetic route is shown in Figure S1. A series of thioester compounds (labeled **1**–**4**) with alkyl chain lengths ranging from 7 to 13 carbons were successfully synthesized, as illustrated in Figure 1A. The NGF-mimic effects of these compounds on PC12 cells were subsequently evaluated. The percentage of PC12 cells exhibiting neurite growth increased in a dose-dependent manner following treatments with compounds **1**–**4** at doses of 0.3, 1, and 3 µM, respectively. Compounds **3** and **4** displayed superior activity in promoting neurite outgrowth (Figure 1B,C).

Additionally, the methyl thiazolyl tetrazolium (MTT) assay was conducted to assess the cytotoxicity of these compounds in PC12 cells. The viability of PC12 cells remained stable after treatment with compounds **1**–**3** at concentrations of 0.1, 1, and 10 µM, indicating low toxicity for these compounds. However, compound **4** demonstrated some level of toxicity at 10 µM (Figure 1D). Consequently, compound **3**, designated as TBG096, was selected for further investigation. The chemical structure of TBG096 was validated by high-resolution electrospray ionization mass spectrometry (HR-ESI-MS), ^1^H NMR, and ^13^C NMR data (Figure S2). To evaluate its safety at the animal level, acute toxicity experiments were performed in mice. These results indicated no significant differences in body weight, food intake, or water consumption following administration (Figure S3A–D). Furthermore, serum biochemical indices, such as aspartate aminotransferase (AST), alanine aminotransferase (ALT), AST/ALT, albumin (ALB), and direct bilirubin (DBIL), showed no significant changes among groups after oral administration of TBG096 at doses of 100, 300, and 600 mg/kg compared with the control group (Figure S3E–I), suggesting that TBG096 does not exhibit acute toxicity in mice at these dosages. Based on the bioactivity and safety of this compound, TBG096 was determined as the lead compound.

### 2.2. TBG096 Exhibits Good Bioavailability and Can Cross the BBB

**Figure 2:**
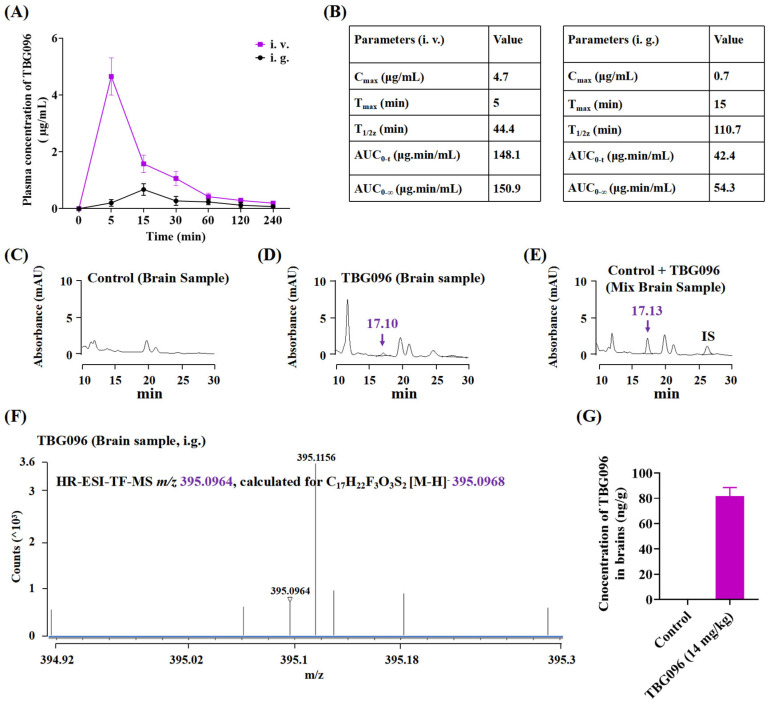
Pharmacokinetic analysis of TBG096 in plasma and brain samples. (**A**) Drug concentration-time curve of TBG096 following a dose of 14 mg/kg by intravenous (i.v.) or oral administration (i.g.) in rats (*n* = 5 rats per group). (**B**) Parameters of TBG096 after intravenous or oral administration in rats. (**C**–**E**) HPLC analysis results of brain homogenate samples in control and TBG096 group (14 mg/kg) after 15 min of single oral administration and HPLC spectra of mixed control sample (including TBG096 and internal standard (IS, compound **4**)). (**F**) HR ESI-TOF-MS spectrometry of collected HPLC peak from brain samples after 15 min of a single oral administration of TBG096 (14 mg/kg). (**G**) The concentration of TBG096 in brain samples following a single dose of 14 mg/kg by oral administration in rats (*n* = 4 rats per group).

Trifluoromethyl (CF_3_) plays an important role in the synthesis of novel drugs. This unique group can prevent metabolism in vivo and improve various pharmacological properties [25]. The introduction of CF_3_, alongside a reduction in the length of the alkyl chain within the structure of this benzoate compound, may enhance its bioavailability. To evaluate the pharmacokinetic properties of TBG096 in vivo, we employed various administration methods to detect its plasma concentration. After intravenous administration of TBG096 (14 mg/kg) to Sprague–Dawley (SD) rats, the highest specific peaks corresponding to TBG096 were detected at 5 min post injection for intravenous administration and 15 min for oral administration at a dose of 14 mg/kg (Figure 2A and Figure S4A,B). The mean area under the curve (AUC_0-∞_) for TBG096 following intravenous injection and oral administration was 150.9 µg·min/mL and 54.3 µg·min/mL, respectively (Figure 2B). The absolute bioavailability of TBG096 was 36.0%, with the oral elimination half-life (t_1/2z_) of 110.7 min.

Additionally, the concentration of TBG096 in the brain was measured at 15 min post oral administration. The results of high-performance liquid chromatography (HPLC) and high-resolution electrospray ionization–time of flight mass spectrometry (HR ESI-TOF-MS) analysis are displayed in Figure 2C–F. Notably, the peak corresponding to TBG096 was detected in both plasma and brain tissues at 15 min following oral administration. The HR ESI-TOF-MS analysis confirmed that the HPLC peak matched the molecular weight of the TBG096 standard. Ultimately, the [M-H]^−^ ion at *m*/*z* 395.0964 for TBG096 was identified in the negative scan mode of the mass spectrometer from the brain sample collected after oral administration (Figure 2F and Figure S4C). The concentration of TBG096 in the brain, 15 min following a single oral dose (14 mg/kg), was calculated to be 81.71 ± 6.807 ng/g (Figure 2G). These results suggest that TBG096 exhibits favorable bioavailability and has the ability to cross the BBB.

### 2.3. TBG096 Produces Neuroprotection and Mitigates Neuroinflammation in HFD-Induced AD Mice

**Figure 3:**
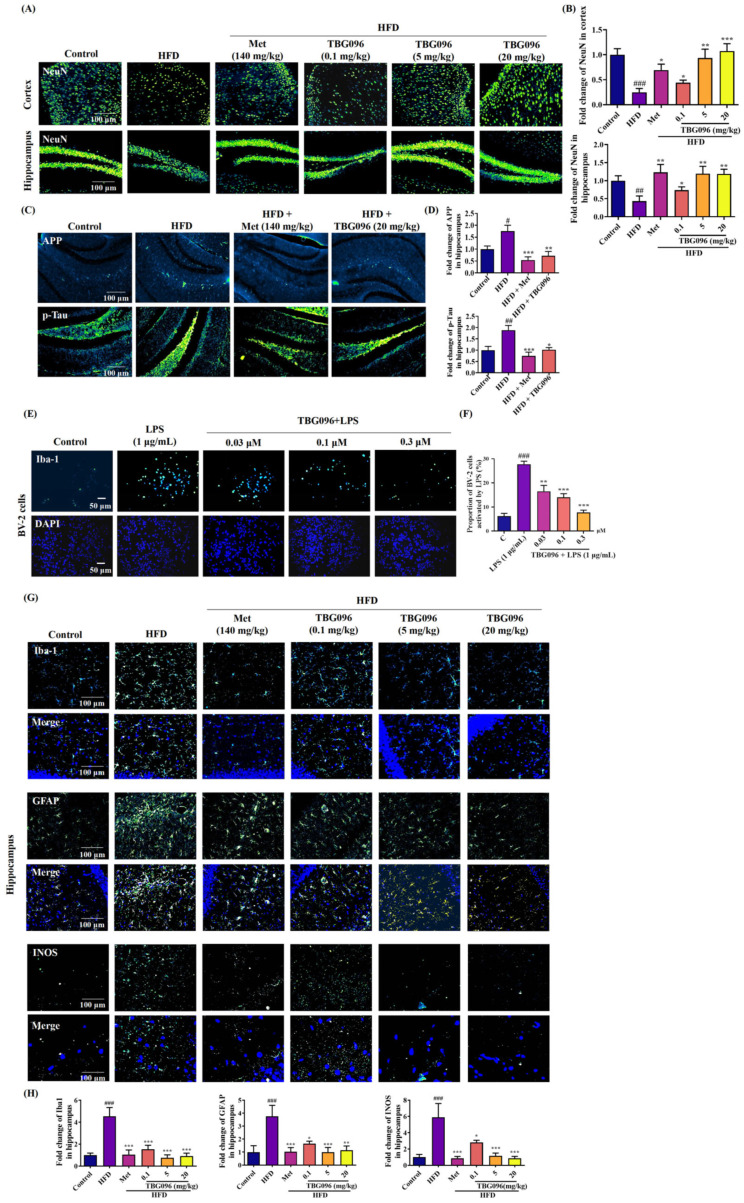
Effects of TBG096 on brain neurons in HFD-induced AD model. (**A**) Fluorescent images and (**B**) digital results of mature neurons in the cerebral cortex and hippocampus of the HFD-induced AD mice. (**C**) Fluorescent images and (**D**) digital results of APP and p-Tau staining in the hippocampus of HFD-induced AD mice. (**E**) Fluorescent images and (**F**) digital results of BV-2 cells after treatment with TBG096 and LPS for 24 h. (**G**) Fluorescent images and (**H**) digital results of Iba1, GFAP, and INOS expressions in the hippocampus of HFD-induced AD mice. The data are presented as mean ± SEM. ^#^, ^##^, and ^###^ indicate significant differences at *p* < 0.05, *p* < 0.01, and *p* < 0.001 compared with the normal control; *, **, and *** indicate significant differences in comparison with the LPS or HFD-only group at *p* < 0.05, *p* < 0.01, and *p* < 0.001, respectively.

One of the characteristics of AD patients is the massive death of neurons in the brain, synaptic loss, and dysfunction, accompanied by the down-regulation of mature neurons. Neuron-specific nuclear protein (NeuN) is expressed in mature neurons of vertebrates. In addition, APP, precursor of Aβ, and high phosphorylation-Tau are biomarkers of AD. To explore the influence of TBG096 on neurons and these biomarkers in the cerebral cortex and hippocampus of AD mice, the immunofluorescence stains of NeuN, APP, and phosphorylation Tau were conducted initially. The number of mature neurons in the HFD-only group was significantly lower than in the control group, whereas TBG096-treated and Met-treated groups showed increased numbers of mature neurons in the cerebral cortex (Figure 3A,B). Meanwhile, the significant increase and reduction in APP and phosphorylation Tau protein in the hippocampus were observed in HFD-only group, Met and TBG096 treated groups, respectively (Figure 3C,D). These results indicate that TBG096 has neuroprotective effects.

Neuroinflammation is closely related to memory dysfunction in AD mice. Inflammation and autophagy of the central nervous system (CNS) are related to the pathology of neurodegenerative diseases. The impact of TBG096 on neuroinflammation was investigated using BV-2 microglial cells and HFD-induced AD mice. TBG096 dose-dependently inhibited lipopolysaccharide (LPS)-induced overactivation of BV-2 cells, with an optimal concentration of 0.3 µM (Figure 3E,F). By detecting the expression of biomarkers including ionized calcium-binding adapter molecule 1 (Iba1), glial fibrillary acidic protein (GFAP), and inducible nitric oxide synthase (INOS), the immunofluorescence staining revealed significantly increased numbers of activated microglia, astrocytes, and M1 macrophages in the hippocampus of HFD-induced AD mice compared to the control, whereas TBG096 treatment significantly reduced these markers (Figure 3G,H). These findings demonstrate that TBG096 mitigates neuroinflammation both in vitro and in vivo.

### 2.4. TBG096 Promotes Neurogenesis in HFD-Induced AD Mice

**Figure 4:**
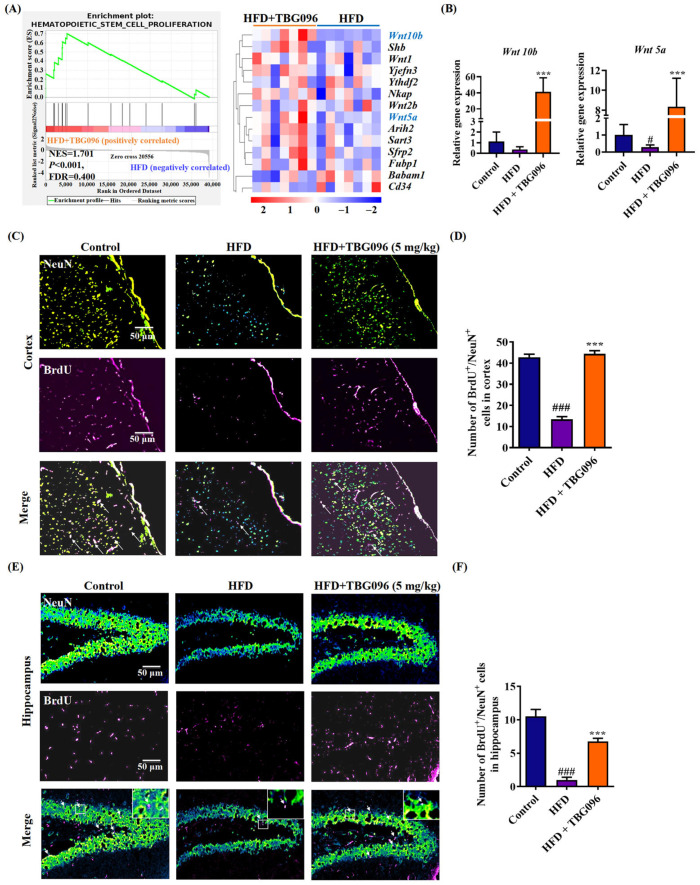
Effects of TBG096 on newborn neurons in HFD-induced AD model. (**A**) GSEA-GO enrichment plot and heat map of the HFD + TBG096 group compared with the HFD-only group regarding gene expression changes in hematopoietic stem cell proliferation in brain samples of the HFD model. The heat map shows the Z-score value distribution of gene expression. Red and blue color represent up- and down-regulation, respectively. (**B**) Relative gene expression level of *Wnt10b* and *Wnt5a* in brain samples of the HFD model. (**C**) Fluorescent images and (**D**) digital results of NeuN and BrdU staining neurons in the cerebral cortex of HFD-induced AD mice. (**E**) Fluorescent images and (**F**) digital results of NeuN and BrdU staining neurons in the hippocampus of HFD-induced AD mice. The white arrows indicate cells that are double-stained with NeuN and BrdU antibodies in (**C**,**E**). The data are presented as mean ± SEM. ^#^ and ^###^ indicate a significant difference at *p* < 0.05 and *p* < 0.001 compared with the normal control; *** indicates a significant difference in comparison with the HFD group at *p* < 0.001.

To further evaluate the effects of TBG096 on the brains of AD mice, transcriptome sequencing was conducted on brain samples from AD mice. The analysis revealed 97 up-regulated and 242 down-regulated genes in the TBG096-treated group compared to the HFD group. Additionally, 391 transcripts were significantly increased, and 537 transcripts were reduced in the TBG096-treated group (Figure S5A–C). A Gene Set Enrichment Analysis (GSEA) of the Gene Ontology (GO) enrichment plot results displayed that the TBG096 group promoted the hematopoietic stem cell proliferation by enhancing the expression of the Wnt gene family (*Wnt10b*, *Wnt5a*, and *Wnt1*) and *Shb*, *Arih2*, *Yjefn3*, *Nkap*, *Sfrp2*, and *Fubp1* (Figure 4A). Quantitative real-time polymerase chain reaction (qRT-PCR) confirmed the role of Wnt signaling in TBG096′s effects by highlighting changes in *Wnt10b* and *Wnt5a* (Figure 4B). The Wnt signaling pathway is critical for neurogenesis and synaptic plasticity and essential for maintaining neuronal connections [10]. Therefore, these results suggest that TBG096 plays an important role in brain neurons of AD mice.

To study the effect of TBG096 on neurogenesis in the cerebral cortex and hippocampus of AD mice, double-staining immunofluorescence analyses of NeuN and bromodeoxyuridine (BrdU) were performed, which are biomarkers for mature neurons and newborn neurons used to detect the changes in the cerebral cortex and hippocampus of AD mice. The number of NeuN^+^ and BrdU^+^ neurons in the HFD-only group was significantly lower than in the control group, whereas TBG096-treated groups showed increased numbers of these neurons in the cerebral cortex (Figure 4C,D). Similarly, the number of newborn neurons in the hippocampus was reduced in the HFD-only group and elevated in the TBG096-treated group (Figure 4E,F). These results indicate that TBG096 induces neurogenesis in the brain of HFD-induced AD mice.

### 2.5. Chaperone-Mediated Autophagy Is Involved in NGF-Mimic and Anti-AD Effects of TBG096 in PC12 Cells and HFD-Induced AD Mice

**Figure 5:**
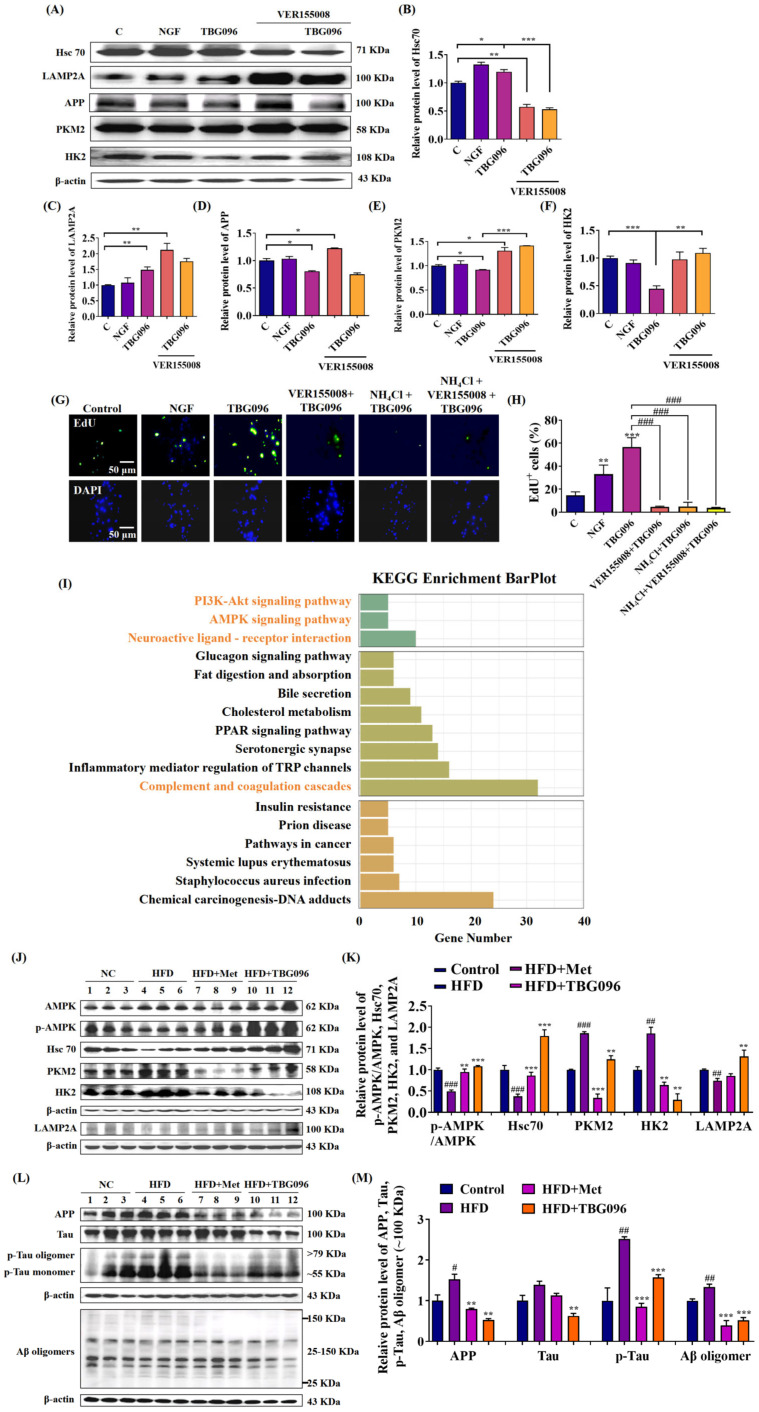
The signaling pathway in which TBG096 works. (**A**) Western blot analysis and (**B**–**F**) digital results of Hsc70, LAMP2A, APP, PKM2, and HK2 proteins in PC12 cells. (**G**) Fluorescent images and (**H**) digital results of EdU and DAPI staining in PC12 cells. (**I**) KEGG enrichment bar plot of the HFD + TBG096 group compared with the HFD-only group for brain tissue. (**J**) Western blot analysis and (**K**) digital results of AMPK, p-AMPK, Hsc70, PKM2, HK2, and LAMP2A proteins in the cerebral cortex of HFD-induced AD mice. (**L**) Western blot analysis and (**M**) digital results of APP, Tau, p-Tau, and Aβ oligomers proteins in the cerebral cortex of HFD-induced AD mice. *, **, and *** indicate significant differences at *p* < 0.05, *p* < 0.01, and *p* < 0.001 in (**B**–**F**). ** and *** indicate significant differences at *p* < 0.01 and *p* < 0.001 compared with the control in (**H**); ^###^ indicate significant differences at *p* < 0.001 in (**H**). ^#^, ^##^, and ^###^ indicate significant differences at *p* < 0.05, *p* < 0.01, and *p* < 0.001 compared with the normal control in (**K**,**M**); ** and *** indicate significant differences at *p* < 0.01 and *p* < 0.001 compared with the HFD-only group in (**K**,**M**).

Previously, we identified Hsc70 as the target protein of one leading compound with a similar structure to TBG096 by the ABPP method [24]. To understand whether Hsc70 is involved in the NGF-mimic effect of TBG096 in PC12 cells, the inhibitor of the Hsc70 protein, VER155008, was used (Figure 5). As expected, the NGF-mimic effect of TBG096 was significantly diminished after treatment with VER155008 (Figure S6A,B). Furthermore, changes in downstream proteins for CMA, such as lysosomal-associated membrane protein 2a (LAMP2A), PKM2, and HK2, were detected after giving the inhibitor. After the treatment of TBG096, the protein levels of Hsc70 and LAMP2A were significantly increased compared with the control group. Meanwhile, the expressions of APP, HK2, and PKM2 protein were significantly decreased. After the treatment of VER155008, the protein expression of Hsc70 was significantly decreased, while those proteins of LAMP2A, APP, and PKM2 were significantly increased, respectively. When PC12 cells were treated with both VER155008 and TBG096, the effects of TBG096 in promoting Hsc70 expression and reducing PKM2 and HK2 expression were abrogated (Figure 5A–F and Figure S7A,B). In addition, we detected the effect of VER155008 (inhibitor of Hsc70) and NH_4_Cl (inhibitor of lysosome) on the EdU cell proliferation activity of TBG096 (Figure 5G,H). It was found that these two inhibitors could reverse the activity of TBG096 in promoting PC12 cell proliferation, respectively. These results indicated that CMA may be involved in the NGF-mimic and neurogenesis effects of TBG096.

Kyoto Encyclopedia of Genes and Genomes (KEGG) pathway enrichment in brain samples of the HFD model suggested that TBG096 was involved in the adenosine 5′-monophosphate-activated protein kinase (AMPK) and PI3K/protein kinase B (AKT) signaling pathway, neuroactive ligand–receptor interaction, inflammatory mediator regulation of TRP channels, and complement and coagulation cascades (Figure 5I). AMPK was identified as an upstream regulator of CMA, which facilitates efficient autophagosome maturation and lysosomal fusion [26], while activation of the PI3K pathway was associated with neurite outgrowth, neuroprotection, and neuroregeneration [11]. To explore the mechanism of action of TBG096 in AD mice, changes in CMA pathway-associated proteins, including AMPK, Hsc70, PKM2, HK2, and LAMP2A, were analyzed in the brains of AD mice. In HFD-induced AD mice, phosphorylated AMPK/AMPK, LAMP2A, and Hsc70 levels were reduced, while HK2 and PKM2 expression levels were elevated. Following TBG096 treatment, these protein levels returned to normal (Figure 5J,K and Figure S7C,D). These results indicate that CMA may also take part in the anti-AD effect of TBG096.

Given that Aβ aggregation and Tau hyperphosphorylation are hallmarks of AD and that APP, the precursor to Aβ, can be degraded via CMA [20], the effects of TBG096 on APP, Tau, p-Tau, and Aβ oligomers were evaluated. The HFD mice had higher protein levels of APP, Tau, p-Tau, and Aβ oligomers in the cerebral cortex, while TBG096 treatment significantly reduced the expression of these proteins in the brains of AD mice (Figure 5L,M and Figure S7E,F), respectively. TBG096 may clear Aβ and p-Tau proteins by regulating the CMA pathway.

### 2.6. TBG096 Improves the Cognitive Dysfunction of HFD-Induced AD Mice

**Figure 6:**
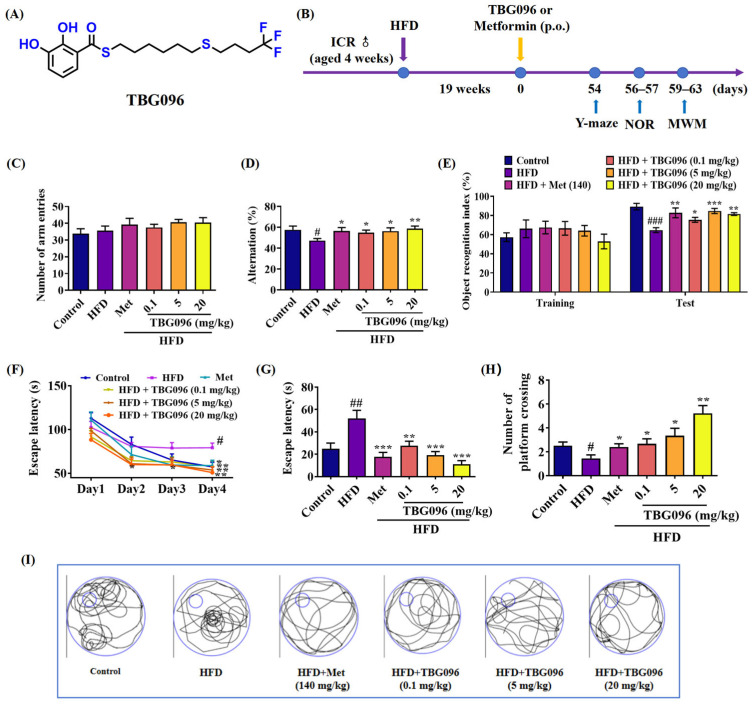
Changes in learning memory and spatial memory of HFD-induced AD mice after administering TBG096. (**A**) The chemical structure of TBG096. (**B**) Schedule of animal experiment for efficacy evaluation of TBG096 in HFD-induced AD mice. (**C**) Changes in the number of arm entries and (**D**) percentage of alternation in the Y-maze test of HFD-induced AD mice. (**E**) Percentage of object recognition index in the NOR experiment of HFD-induced AD mice. (**F**) Escape latency in the training phase of MWM test of HFD-induced AD mice. (**G**) Escape latency and (**H**) platform crossing numbers in the test phase of the MWM test of HFD-induced AD mice. (**I**) Images of platform crossing trajectory. The big circle represents the pool of the water maze, the small circle represents the platform position, and the curve represents the swimming exploration trajectory. The data are presented as mean ± SEM (*n* = 10 mice per group). ^#^, ^##^, and ^###^ indicate significant differences in comparison with the control group at *p* < 0.05, *p* < 0.01, and *p* < 0.001. *, **, and *** indicate significant differences in comparison with the HFD group at *p* < 0.05, *p* < 0.01, and *p* < 0.001, respectively.

Metabolic dysfunction is associated with impaired cognitive function and an elevated risk of age-related cognitive decline (ACD) and AD, usually resulting from diets high in saturated fats and sugars [27]. To assess the potential anti-AD effects of TBG096 (chemical structure in Figure 6A) at the animal level, a mouse model of AD induced by the HFD for drug efficacy evaluation was initially utilized. The experimental scheme is shown in Figure 6B. Behavioral experiments were conducted to examine the learning and memory behaviors of the AD model mice (Figure S8A–F), confirming the successful establishment of the HFD-induced AD mouse model. Metformin (Met) has been reported to activate Hsc70-mediated autophagy and alleviate the pathology in mouse models of AD [20]; thus, it was employed as a positive control in our HFD-induced AD model. Following administration of TBG096 at doses of 0.1, 5, and 20 mg/kg, along with Met at a dose of 140 mg/kg for 54 days, animal behavior tests were performed to detect changes in memory of HFD-induced AD mice. No significant differences were observed in the number of arm entries across all groups during the Y-maze test (Figure 6C). However, the alternation behavior in the HFD-only group was significantly lower compared to the normal control group. In contrast, after treatment with TBG096 at all doses (0.1, 5, and 20 mg/kg), the alternation behavior in AD mice was significantly improved, with the 20 mg/kg dose demonstrating the most pronounced effect (Figure 6D). In the novel object recognition (NOR) test, no significant differences were noted in the discrimination index during the training phase. However, during the test phase, the discrimination index for the TBG096-treated HFD mice was higher than that of the HFD-only group, with an optimal dose identified as 5 mg/kg (Figure 6E).

Additionally, the Morris water maze (MWM) test evaluated the spatial long-term memory of mice. In the training phase, the escape latency for AD mice in the HFD-only group significantly increased by the fourth day compared to the normal control group. Conversely, this latency was markedly reduced in AD mice following treatment with Met and all doses of TBG096 (Figure 6F). On the fifth day of the test phase, significant reductions in escape latency were also noted in the TBG096 treatment groups (Figure 6G), and the number of platform crossings increased significantly in both Met and TBG096 treatment groups when compared to the HFD-only group (Figure 6H,I). These results indicate that TBG096 could improve the working memory, short-term memory, and long-term memory of HFD-induced AD mice, with the optimal therapeutic effect at a dosage of 20 mg/kg, which is comparable to that of Met.

## 3. Discussion

Previous studies identified gentiside derivatives with novel NGF-mimic effects. However, these compounds exhibited low bioavailability and poor ability to cross the BBB. To address these limitations, this study introduced structural modifications, including alterations to alkyl chain length and substitution of the terminal methyl group’s hydrogen atom with fluorine (Figures S1 and S2). These modifications led to the development of thiobenzoate compounds with improved water solubility (Figure 1), and TBG096 was discovered as a lead compound with NGF-mimic activity and favorable safety (Figure 1 and Figure S3).

As a potential therapeutic candidate for neurodegenerative diseases, TBG096 required good bioavailability and BBB permeability. Pharmacokinetic studies assessed its brain tissue concentration, while HPLC and HR ESI-TOF-MS analysis confirmed that TBG096 exhibited favorable bioavailability and successfully crossed the BBB (Figure 2 and Figure S4). NGF cannot penetrate the BBB to produce a therapy effect due to high molecular weight and polarity [28]. TBG096 and Met can avoid this limitation, pass through BBB [29], and arrive at the brain tissue to exert anti-AD effects. However, Met mainly acts on the liver and intestine [30]. Interestingly, the oral bioavailability and the half-life of TBG096 and metformin are 36% and 1.85 h as well as 55% and 1.92 h [31,32], respectively. However, TBG096 has anti-AD effects at low concentrations of 0.1 and 5 mg/kg. These findings suggest that TBG096 is a highly promising candidate drug for anti-AD treatment.

Key pathological features of AD include the loss of neurons [33]. To evaluate the effects of TBG096 on neuronal integrity, immunofluorescence staining was performed on the cerebral cortex and hippocampus of AD mice. The analysis revealed marked reductions in mature neurons, increased APP, and phosphorylated Tau proteins due to long-term HFD feeding, which were notably reversed following TBG096 treatment, indicating that TBG096 has a neuroprotective effect (Figure 3). Neuroinflammation is another major contributor to AD pathology, driven primarily by astrocytes and microglia [34]. The effects of TBG096 on microglial and astrocytic activity as well as INOS levels were assessed (Figure 3).

Reducing Aβ deposition has been a primary therapeutic focus for AD, but evidence supporting its ability to halt disease progression remains inconclusive. Recently, neuronal regeneration has been gradually accepted, and emerging evidence has reported that blocking the adult hippocampal neurogenesis (AHN) exacerbated cognitive impairment in AD mice, which exhibit typical AD pathological features such as Aβ aggregation; similar changes in AHN were also found in AD patients [35]. Furthermore, combining neurogenesis with brain-derived neurotrophic factor (BDNF) has been shown to replicate the cognitive benefits of exercise in AD models [3]. To explore the role of neurogenesis in the anti-AD effects of TBG096, the sequencing analysis of transcriptome and immunofluorescence staining of newborn neurons in the hippocampus and cortex of HFD mice was evaluated (Figure 4 and Figure S5). These experiments show that Wnt signaling is involved in the function of TBG096. Interestingly, treatment with TBG096 increased the number of newborn neurons in both regions. Although neurogenesis is traditionally associated with the hippocampus, studies have reported potential neurogenic activity in the cortex of adult non-human primates [36]. Neurogenic activity observed in the cerebral cortex of TBG096-treated mice warrants further investigation to determine its extent and possible migratory behavior within the brain. These results confirmed that TBG096 promotes neurogenesis and mitigates neuroinflammation, contributing to its anti-AD effects.

CMA is a selective autophagic process that degrades cytosolic proteins containing KFERQ motifs through lysosomal pathways [16]. To indicate whether CMA participates in the NGF mimic and anti-AD effects of TBG096, the inhibitor experiment and Western blot analysis were conducted. The results in Figure 5, Figures S6 and S7 clarify that TBG096 exerted NGF-mimic activity in PC12 cells and anti-AD effect by activating Hsc70 and regulation downstream of CMA. The APP protein as one of the substrates of CMA is responsible for generating Aβ peptides, which subsequently aggregate to form senile plaques in the brain [20]. Concurrently, the Tau protein, critical for stabilizing microtubules, undergoes hyperphosphorylation in AD, contributing to neuroinflammation, synaptic dysfunction, and cognitive decline [37]. Thus, the effects of TBG096 on APP, Aβ, Tau, and p-Tau levels were evaluated. Significant reductions in these proteins after TBG096 treatment, as shown in Figure 5 and Figure S7, suggest that CMA may take part in the clearance of Aβ and p-Tau in AD mice of TBG096. In the present study, we only clarify that TBG096 produces anti-AD effects via promoting neurogenesis and modification of CMA. Whether CMA is involved in the neurogenesis of TBG096 needs more evidence to confirm.

TBG096 was evaluated in the HFD-induced AD mice—selected for its close resemblance to human AD pathology. The behavioral assessments in Figure 6 and Figure S8 revealed that TBG096 at all doses of 0.1, 5, and 20 mg/kg exerted anti-AD effects in this model in a dose-dependent manner, and the optimal dose for TBG096 was 20 mg/kg, with comparable efficacy to Met at a concentration of 140 mg/kg. These results align with findings for other compounds such as CuB and 2H-GPS, which utilize different mechanisms to exert anti-AD effects [38,39]. CuB primarily acts through neurogenesis and neuroprotection, while 2H-GPS regulates the Wnt signaling pathway. In contrast, TBG096 exerts NGF-mimic activity in PC12 cells and addresses memory dysfunction in AD by promoting neurogenesis and regulation of the Hsc70 signaling pathway.

Additionally, we discovered a compound similar to TBG096 modulating the abundance of gut microbiota in previous studies [40]. Hence, we examined TBG096′s impact on gut microbiota in HFD-induced AD mice. In HFD mice, the abundance of the beneficial gut bacteria *Ligilactobacillus* was significantly decreased but improved with TBG096 (5 and 20 mg/kg) or Met (140 mg/kg) treatment (Figure S9A). The principal coordinate analysis (PCoA) showed HFD altered gut microbiota composition, which TBG096 reversed towards normal levels, with 20 mg/kg closest to the control group (Figure S9B). The α diversity analysis revealed significant differences between TBG096 (0.1 mg/kg) and HFD groups, with TBG096′s goods coverage near the control group (Figure S9C). Phylum composition analysis showed the abundance of *Firmicutes* was decreased and *Bacteroidota* increased in the HFD group, but this was reversed with TBG096 (20 mg/kg) and Met (140 mg/kg) treatment towards normal levels (Figure S9D). Thus, TBG096 may reshape gut microbiota by enhancing *Ligilactobacillus* and normalizing *Firmicutes* and *Bacteroidota*, which are dominant in the intestinal microbial community. Clinical studies have shown that gut microbiota is related to the risk of AD [41]. Changes in gut microbiota can increase the risk of AD by promoting neuroinflammation. In subsequent research, we will conduct an in-depth study on the relationship between specific gut microbiota modulated by TBG096 and neuroinflammation.

**Figure 7:**
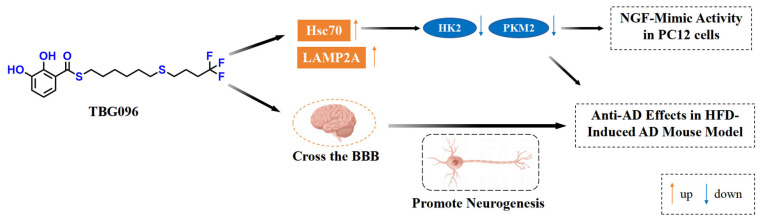
The potential mechanism of action of TBG096. TBG096 promotes neurogenesis and regulates the Hsc70 signaling pathway to exert NGF-mimic activity and produce anti-AD effects in AD mice.

In conclusion, this study confirms TBG096 as a promising candidate drug for AD. This molecule has good bioavailability and can cross the BBB. TBG096 exerted NGF-mimic activity by Hsc70 and produced anti-AD effects in HFD-induced AD mice via promoting neurogenesis (Figure 7). In the future, we will examine the potential limitations of TBG096, such as its long-term safety, impact on non-neuronal cells, and synergistic side effects with other AD treatment strategies. Meanwhile, we will focus on identifying the binding targets and binding sites of TBG096 and verify whether these proteins are indeed therapeutic targets responsible for the anti-AD effects of TBG096.

## 4. Materials and Methods

### 4.1. General

Silica gel (200–300 mesh, Yantai Research Institute of Chemical Industry, Yantai, China) and reversed-phase C18 (octadecylsilyl, ODS) silica gel (Cosmosil 75 C18-OPN, Nacalai Tesque, Kyoto, Japan) were used for column chromatography. Precoated silica gel (0.25 mm and RP-18 plate (0.25 mm; Merck KGaA, Darmstadt, Germany)) was used for TLC. The preparative HPLC was performed on an HPLC system equipped with ELITE P-230 pumps (Dalian Elite Inc., Dalian, China). NMR spectra were recorded on a Bruker AV III-500 spectrometer (Bruker, Karlsruhe, Germany). High-resolution (HR) ESI-TOF-MS were recorded on an Agilent 6224A LC/MS (Agilent Technologies Inc., Beijing, China).

### 4.2. Reagents and Antibodies

The sources of chemicals used in experiments are as follows: DMSO (#D4540), NGF (#N2513), LPS (#L2880), metformin (#317240), and BrdU (#B5002) were purchased from Sigma-Aldrich (St. Louis, MO, USA); VER155008 (#HY-10941) was purchased from MedChemExpress (Shanghai, China); NH_4_Cl (#A116372) was purchased from Aladdin Biochemical Technology (Shanghai, China). The following antibodies were used in this study: HK2 (#22029-1-AP), PKM2 (#15822-1-AP), APP (#60342), and Tau (#66499-1-Ig) from Protein Tech (Chicago, IL, USA); LAMP2A(#ET1601-24) from HuaAn Biotechnology (Hangzhou, China); Aβ (#8243S), AMPK (#2532), and p-AMPK (#2535) from Cell Signaling Technology (Boston, MA, USA); NeuN (#ab177487), BrdU (#ab8152), Hsc70 (#ab51052), p-Tau (phospho S396) (#ab32057), Iba1 (#ab5076), GFAP (#ab7260), INOS (#ab178945), and goat anti-rabbit IgG H&L (Alexa Fluor^®^ 488, #ab6702) from Abcam (Cambridge, UK); rabbit anti-mouse IgG (Cy3 conjugate) from Millipore (Merck KGaA, Darmstadt, Germany); and β-actin (#CW0096M) and secondary antibody horseradish peroxidase [HRP]-linked anti-rabbit and anti-mouse IgGs (#CW0103) from CoWin Biotech Company (Beijing, China).

### 4.3. Synthesis and Purification of TBG096

The synthesis of TBG096 (compound **3**) was used as an example, and the synthesis and purification procedures of TBG096 were undertaken as follows. At first, 1,6-Hexanedithiol (3.01 g, 20.00 mmol) was dissolved in anhydrous N,N-dimethylformamide (DMF) (50 mL). After that, 60% NaH (1.8 g, 45 mmol) was added at 0 °C, kept stirring for 30 min, and warmed up to room temperature for another 30 min. Subsequently, 1-bromo-4,4,4-trifluorobutane (3.0 mL, 20.00 mmol) and tetrabutylammonium iodide (TBAI) (740 mg, 2.00 mmol) were added in at 0 °C. Then, the mixture was stirred and warmed up to room temperature overnight. The reaction was stopped by adding 500 mL of EtOAc. The mixture was washed with 1 N HCl solution, water, and saturated aqueous solution of NaHCO_3_ and NaCl, successively. The organic phase was dried over Na_2_SO_4_, filtered, and then concentrated. Next, the product of the first step was dissolved in anhydrous CH_2_Cl_2_ (50 mL), and DMAP (2.44 g, 20.0 mmol), EDC·HCl (3.82 g, 20.0 mmol), and 2,3-dihydroxybenzoic acid (1.54 g, 10.00 mmol) were added into this mixture. The mixture was stirred overnight at room temperature and then concentrated under vacuum. The crude mixture was purified by silica gel open column (*n*-hexane: EtOAc = 80:1) and ODS open column (Methanol: H_2_O = 80:20) and then purified by HPLC (SP-ODS-A (20 × 250 mm), MeOH/H_2_O = 87:13 in 40 min, flow rate: 8 mL/min, detected at 254 nm) to obtain a pure compound **3** (TBG096, tR = 28 min, 5 injections, 249 mg, yield 6.3%).

The structure of TBG096 was determined by ^1^H NMR, ^13^C NMR, and HR ESI-MS data. ^1^H NMR (500 MHz, CDCl_3_): *δ* = 11.24 (1H, s), 7.41 (1H, dd, *J* = 1.4, 8.0 Hz), 7.11 (1H, dd, *J* = 1.4, 8.0 Hz), 6.82 (1H, t, *J* = 8.0 Hz), 5.67 (1H, s), 3.07 (2H, t, *J* = 7.3 Hz), 2.55 (4H, m), 2.22 (2H, m), 1.85 (2H, m), 1.70 (2H, m), 1.60 (2H, m), 1.45 (4H, m). ^13^C NMR (125 MHz, CDCl_3_): *δ* = 197.9, 146.8, 145.4, 128.3 (q, ^1^*J* (C, F) = 276.3 Hz), 120.1, 119.9, 119.5, 77.4, 32.6 (q, ^2^*J* (C, F) = 28.6 Hz), 31.9, 31.0, 29.5, 29.3, 28.8, 28.5, 28.4, 22.0 (q, ^3^*J* (C, F) = 2.6 Hz). HR ESI-TOF-MS *m/z* 419.0933, calcd. for C_17_H_23_F_3_O_3_S_2_Na [M + Na]^+^ 419.0933. The synthesis and purification procedures of compounds **1**, **2**, and **4** and other details are presented in the Supporting Information (Sections S1.1 and S1.2; Figures S1 and S2).

### 4.4. Cell Culture and Activity Evaluations for Neurogenesis and Anti-Inflammation

PC12 cells were purchased from the Cell Bank of the Chinese Academy of Sciences (Shanghai, China) and cultured in DMEM medium (Dulbecco’s Modified Eagle Medium, CellMax, Lanzhou, China), supplemented with 10% HS (Horse Serum, Solarbio, Beijing, China), 7.5% FBS (Foetal Bovine Serum, CellMax, Lanzhou, China), and 1% penicillin/streptomycin (Solarbio, Beijing, China).

The neurogenic activity of samples was evaluated according to the methods described in our previous study [24]. Briefly, approximately 5 × 10^4^ cells were seeded in each well of a 24-well microplate and were cultured under a humidified atmosphere with 5% CO_2_ at 37 °C for 24 h. Then, the medium in each well was replaced with 1 mL of serum-free DMEM containing 40 ng/mL NGF as a positive control, 0.5% DMSO as a negative control, and the test sample after 24 h, respectively. About 100–200 cells of each well were counted from a randomly selected area and repeated thrice after 48 h. A cell-bearing neurite outgrowth longer than the diameter of the cell body was identified as a positive cell. The activity was presented as the percentage of positive cells in the selected area.

BV-2 cells were obtained from Nanjing CoBioer Biotechnology Ltd., Co., Nanjing, China, and cultured in RPMI 1640 medium (Roswell Park Memorial Institute 1640 medium, Gibco, Grand Island, NY, USA) supplemented with 10% FBS and 1% penicillin/streptomycin. The anti-inflammatory activity of samples in BV-2 cells was determined as follows. Approximately 5 × 10^4^ cells were seeded in each well of a 24-well microplate and were cultured under a humidified atmosphere with 5% CO_2_ at 37 °C for 24 h. Then, the medium was replaced by 500 µL RPMI 1640 medium containing 0.5% DMSO or samples at different concentrations for 2 h, and 500 µL medium containing LPS (1 µg/mL) was added and incubated for 24 h. Next, the medium of each well was sucked out, and each well was washed with phosphate-buffered saline (PBS) three times and fixed with 4% paraformaldehyde (500 µL/well) for 20 min at room temperature. The fixative was discarded, washed with PBS, and blocked for 60 min. After removing the blocking solution, the primary antibody (Anti-Iba1 rabbit monoclonal antibody) was diluted 1000 times, and 200 µL of diluted primary antibody was added to each well and then incubated at 4 °C overnight. The next day, the wells were washed with PBS, and goat anti-rabbit IgG (250 µL/well) diluted 1000 times was added and incubated at room temperature for 1 h. After that, the cell nucleus was stained with diluted DAPI (SouthernBiotech, Birmingham, AL, USA) for 10 min, and the fluorescence image of BV-2 cells was observed under the fluorescence inverted microscope. Three regions were randomly selected to be observed and recorded with pictures. Image J software version 1.8.0 (National Institutes of Health, Bethesda, MD, USA) was finally used to analyze the fluorescence density of BV-2 cells.

### 4.5. Methyl Thiazolyl Tetrazolium (MTT) Assay

PC12 cells were firstly inoculated into a 24-well plate and cultured for 24 h. Following this, samples at different doses were added and cultured for 24 h. Subsequently, the 0.5 mL serum-free medium containing MTT (0.2 mg/mL) of each well was used to replace the original medium and incubated for 2 h. After removing the solution, the purple crystal was dissolved in 200 μL of DMSO, and the absorbance was measured at 570 nm. The cell survival rate for each group was calculated based on the control group.

### 4.6. Inhibitor Experiment

In the inhibitor experiment of NGF-mimic activity, approximately 5 × 10^4^ PC12 cells were seeded into each well of a 24-well plate and incubated for 24 h. After that, the medium in each well was replaced with 500 μL of serum-free DMEM or the serum-free DMEM containing VER155008, the inhibitor of Hsc70 protein, incubated in a CO_2_ incubator for 30 min. Subsequently, 500 μL of serum-free DMEM containing different samples was added and cultured, respectively. The PC12 cells with neurite outgrowth were recorded after 48 h. For the Western blot analysis, the experiment included five groups, namely the control (C) group, the NGF (40 ng/mL) group, the TBG096 (1 µM) group, the VER155008 (3 µM) group, and the VER155008 (3 µM) + TBG096 (1 µM) group. Afterwards, approximately 2 × 10^6^ cells per dish were seeded into ten 6 cm Petri dishes with 5 mL of DMEM medium and incubated for 24 h. After giving different treatments with samples, the cells continued to culture for 4 h. Protein extraction was conducted to prepare for the subsequent Western blot analysis.

In the inhibitor experiment of EdU cell proliferation activity, PC12 cells were seeded into each well of a 24-well plate as above for 24 h. Then, the medium in each well was replaced with 500 μL of serum-free EM containing VER155008 (inhibitor of Hsc70, 3 µM), or NH_4_Cl (inhibitor of lysosome, 250 µM) for 30 min according to the literature [24,42], and 500 μL of serum-free DMEM containing different samples was added and cultured for 24 h, respectively. After that, PC12 cells were treated with EdU for 2 h according to the operation steps of the BeyoClick™ EdU-488 cell proliferation detection kit (#C0071S, Beyotime Biotechnology, Shanghai, China). After cell labeling, the supernatant was removed. Then, PC12 cells were fixed, washed, and blocked, and the click reaction solution was added at room temperature for 30 min. After that, DAPI staining was performed for 10 min. Finally, PC12 cells were detected by fluorescence microscope (Olympus IX53, Tokyo, Japan).

### 4.7. Design of Animal Experiments

In experiments with the HFD-induced AD mouse model, a total of 60 Institute of Cancer Research (ICR) male mice aged 4 weeks from Zhejiang Academy of Medical Sciences (Hangzhou, China) were used for animal experiments (permit number: ZJU20230121). The animals were provided free access to a chow diet and water. After one week of acclimatization, ten mice were fed a normal chow diet, whereas the remaining 60 mice were fed the HFD (24.2% crude protein, 42.1% carbohydrate, and 25.4% fat; Biotechnology HD Co., Ltd., Beijing, China). The HFD and normal diet were alternated every two days to maintain freshness. Weekly measurements were taken for water intake, food intake, and body weight. In the eighteenth week, the mice underwent Y-maze and NOR tests. The Morris water maze test was conducted during the nineteenth week. After confirming the successful establishment of the AD model characterized by cognitive impairment, oral administration of treatments commenced. The mice were randomly divided into the following groups: (i) control group, (ii) HFD-only group, (iii) HFD + Met 140 mg/kg group, (iv) HFD + TBG096 0.1 mg/kg group, (v) HFD + TBG096 5 mg/kg group, and (vi) HFD + TBG096 20 mg/kg group. Each group had 10 mice. Soybean oil and MilliQ water were used as vehicles to dissolve TBG096 and Met. The drugs were orally administrated to mice once a day according to the different doses of TBG096 and Met, and this animal experiment was conducted for three months.

In order to investigate the effect of TBG096 on promoting neuronal regeneration, animal experiments were carried out according to the method previously reported [38]. Fifteen 4-week-old ICR male mice purchased from Zhejiang Academy of Medical Sciences (Hangzhou, China) were randomly divided into three groups, namely the (i) normal control group, (ii) HFD group, and (iii) HFD + TBG096 5 mg/kg group, and given a normal diet or high-fat diet for two months to construct the AD mouse model, respectively. After confirming the successful construction of the AD model, TBG096 was administered once a day at a dose of 5 mg/kg for 1 month, and soybean oil was used as a vehicle. BrdU was then injected into the mice at a dose of 100 mg/kg in four injections over two days, each 12 h apart. Administration of TBG096 was continued for 28 days. At the end of animal experiments, the mice were anesthetized and cardiac perfusion stopped. The brains of mice were taken, fixed, and sectioned, and immunostaining was performed with NeuN and BrdU antibodies.

In the acute toxicity experiment of mice, a total of 40 ICR male mice aged 8 weeks from Zhejiang Academy of Medical Sciences (Hangzhou, China) were used. A standard experimental scheme was adopted according to the requirements of Zhejiang University Animal Experiment Ethics Committee (animal use license number: SYXK(Zhe)2019-0002, permit number: ZJU20230219). The mice were randomly divided into the following groups: (i) control group, (ii) TBG096 100 mg/kg group, (iii) TBG096 300 mg/kg group, and (iv) TBG096 600 mg/kg group. Each group had 10 mice. Soybean oil was used as a vehicle to dissolve TBG096. The body weight, food intake, and water consumption of mice in each group were recorded every day for two weeks. Blood was collected for biochemical index analysis.

### 4.8. The Pharmacokinetic Analysis of TBG096

In the pharmacokinetic analysis, a total of 23 female SD rats aged 8 weeks from Zhejiang Academy of Medical Sciences (Hangzhou, China) (permit number: ZJU20240191) were used. A total of 15 rats were randomly divided into the following groups: (i) control group, (ii) TBG096 (14 mg/kg) oral administration group, and (iii) TBG096 (14 mg/kg) intravenous administration group. TBG096 was administered orally (dissolved in soybean oil) or intravenously (dissolved in Milli-Q water containing 2% Tween 80 and 5% ethanol) at 14 mg/kg after 12 h of fasting. Then, the blood from the posterior ocular venous plexus of rats was taken at 5, 15, 30, 60, 90, 120, 180, and 240 min after administration. A total of eight rats were randomly divided into two groups for taking brain samples: (i) control group and (ii) TBG096 (14 mg/kg) oral administration group.

The collected blood was placed in a centrifuge tube (containing 20 µL heparin sodium solution) and centrifuged at 3000 rpm for 10 min to obtain a supernatant (plasma). Brain samples were collected after the oral administration of 14 mg/kg TBG096 and heart perfusion with PBS. Then, 10 µL of 10% formic acid and 10 µL of internal standard (10 ng/µL) were added to 200 µL of plasma (compound **2** was used as internal standard) or 0.2 g of brain sample (compound **4** was used as internal standard) and vortexed for 10 s and then left at −80 °C overnight. Next, 600 µL or 3 mL of extraction solvent (isopropanol) was added to plasma or brain samples, vortexed for 5 min, left at −30 °C for 2 h, and then centrifuged at 12,000 rpm for 15 min. Next, the upper organic phase was transferred to another tube, centrifuged, and concentrated to dryness. The residue was dissolved in 100 µL of mobile phase, vortexed for 0.5 min, and centrifuged at 12,000 rpm for 3 min, and the supernatant was then prepared for HPLC analysis (Supersil Phenyl 5 µm (4.6 × 200 mm), elution with MeCN/H_2_O containing 0.1% formic acid (plasma samples: 57% MeCN/H_2_O, brain samples: 56% MeCN/H_2_O) for 30 min, flow rate: 1 mL/min, sample size: 20 µL, detected at 284 nm) and HR ESI-TOF-MS analysis.

### 4.9. Animal Behavioral Tests

#### 4.9.1. Y-Maze Test

The Y-maze test is used to evaluate working memory, and the process was referenced in another report [39]. In brief, the Y-maze consists of three black plastic arms (A, B, and C) with the same length (30 cm), width (10 cm), height (15 cm), and 120° relative to adjacent arms. Each mouse is placed facing the wall of arm A and allowed to explore the maze freely for 5 min. The behavior of mice is recorded with a video recorder, and the data are stored in ANY-MAZE software version 6.35 (Stoelting, Chicago, IL, USA). The total number of entries made by the mice into each arm—considered as either whole or half-body entries—is recorded. An alternation is defined as a mouse moving through all three arms without returning to the initial arm on the third visit. Alternation: number of alternations/(number of arm entries − 2) × 100%.

#### 4.9.2. Novel Object Recognition (NOR) Test

The NOR test is utilized to assess the working memory capabilities of mice. The NOR apparatus consists of an open, black plastic box, two identical objects, and one novel object. The activity of the mice within the open field is captured and recorded using a video recorder, with data subsequently stored in ANY-MAZE software. The test is conducted over a span of two days, incorporating habituation, training, and testing stages. During the habituation phase, the mice are placed in the box devoid of any objects, allowing them to explore freely for a duration of five min. Following a 24 h interval, the NOR test is administered, which consists of both the training and testing phases. In the training phase, each mouse is positioned in the central area of the box, where two identical objects are placed equidistant from the mouse. Each training session lasts for five minutes. After a one-hour break, one of the identical objects is replaced with a new, distinct object, and the exploration duration for each mouse is recorded. Exploration is defined by the time a mouse spends touching or sniffing an object. The exploration times during both the training and testing phases are documented via video recording, and the data are organized within ANY-MAZE software. The object recognition index was defined as follows: (time spent on the new object)/(time spent on the old object + time spent on the new object) × 100%. The data are calculated by ANY-MAZE software.

#### 4.9.3. Morris Water Maze (MWM) Test

The MWM test is used to measure spatial memory and long-term memory. The whole MWM equipment includes a circular water tank (diameter 1.5 m, depth 50 cm) with warm water (22 ± 1 °C) 35 cm deep, a platform (diameter 14 cm, immersed 1 cm in water), a camera, and a computer for data analysis. The MWM test is conducted over five 5 days: 4 days for training and 1 day for testing. The pool is divided into four quadrants, and at least three quadrants are tested at a time. In the training stage, the mouse is placed on the wall of one of the four quadrants and allowed to swim and find the platform hidden underwater within 120 s. If the mouse cannot find the platform, the experimenter will guide it and let it stay on the platform for 10 s. The experiment lasts for 4 days. On the fifth day of the test phase, the platform is removed, and each mouse is placed in a similar starting position and allowed to move freely for 90 s. The swimming trajectories are recorded and stored in the computer. The time taken by each mouse to reach its destination (escape delay) and the number of times the mouse passes through the platform are recorded by the camera, respectively, and analyzed using ANY-MAZE software.

### 4.10. Immunostaining of Brain Tissues

At the end of animal experiments, three mice in each group were taken for heart perfusion with cold PBS and 4% paraformaldehyde. After that, the brains of mice were fixed with 4% paraformaldehyde for 24 h and gradually dehydrated with 15% and 30% sucrose solutions for 48 h, respectively. The fixed and embedded brain sample was placed in a cryostat and cut into 20 µm thick slices with a cryostat (Thermo Fisher, Waltham, MA, USA), and brain slices in the hippocampus and cortex were collected. Finally, the brain slices were placed in antifreeze (glycerol/ethylene/PBS = 3:3:4) and kept in a refrigerator at −30 °C until immunostaining. The brain slices were fixed on a glass slide and hydrated with PBS for 5 min. Then, blocking buffer was added to the brain slices and incubated at room temperature for 1 h. Brain slices were washed twice with PBS and incubated with anti-NeuN, anti-BrdU, anti-Iba1, anti-GFAP, anti-INOS, anti-APP, or anti-p-Tau at 4 °C for 24 h, respectively. Subsequently, the brain slices were incubated with secondary antibody (goat anti-rabbit IgG H&L (Alexa Fluor^®^ 488) or rabbit anti-mouse IgG (Cy3 conjugate)) at room temperature for 2 h after washing with PBS three times. Finally, a cover glass was used to lightly cover the slices stained with DAPI. The slides loaded with brain sections were observed with an upright two-photon confocal microscope (Olympus BX61, Tokyo, Japan). Biomarker proteins in the cerebral cortex and hippocampus were digitized by Image J software (National Institutes of Health, Bethesda, MD, USA).

### 4.11. Western Blot Analysis

Approximately 50 mg of cerebral cortex from brain tissues was homogenized in RIPA lysis buffer containing 1% protease inhibitor and 1% phosphatase inhibitor. Then, the sample was centrifuged at 12,000 rpm at 4 °C for 10 min, and the supernatant was transferred to a new tube. Protein concentrations were determined using the BCA kit, and 30 µg of protein from each sample was denatured by heating in water at 100 °C for 10 min. Subsequently, approximately 30 µg of protein from each sample was loaded into each well. After conducting gel electrophoresis at 80 V for 15 min and then at 120 V for 60 min, the proteins were transferred to the polyvinylidene fluoride (PVDF) membrane for 90 min. Then, the PVDF membrane was blocked with 5% skimmed milk at room temperature for 60 min. The membrane was incubated with a diluted primary antibody at 4 °C overnight. After washing the membrane three times with TBST buffer (1× TBS buffer containing 0.05% (*v*/*v*) tween 20), it was incubated with the secondary antibody for 1 h at room temperature. Finally, the strip was developed using chemiluminescence ECL reagent, and the blot density was quantified with ImageJ (National Institutes of Health, Bethesda, MD, USA).

### 4.12. Quantitative Real-Time Polymerase Chain Reaction (qRT-PCR)

Total RNA was extracted and quantified from 50 mg of the cerebral cortex using TRIzol reagent (Invitrogen, Carlsbad, CA, USA) and NanoDrop (Thermo Fisher Scientific, Waltham, MA, USA), respectively. Subsequently, 2.5 µg of RNA from each sample was reverse-transcribed using a HiFi-MMLV cDNA kit (Cowin Biotechnology Company, Beijing, China). Transcripts were quantified by PCR analysis performed with a CFX 96 Touch (Bio-Rad, Hercules, CA, USA) and SYBR Premixed EX Taq (Takara, Otsu, Japan). PCR was performed under the following conditions: 95 °C for 2 min, followed by 40 cycles, 94 °C for 15 s, 60 °C for 25 s, and 72 °C for 10 s. All results were normalized to the level of 18S RNA, and the relative mRNA transcription levels were estimated by 2^−ΔΔCT^. The number of animal samples in each group was five, and all samples were run in triplicate, and their average values were calculated. The mouse primers of *Wnt10b* (NM_011718.2), *Wnt5a* (NM_001256224.2), and *18S* RNA (NR_003278.3) were synthesized by Sangon Biotech Co., Ltd. (Shanghai, China), and their sequences are as follows: *Wnt10b*-sense: 5′-AATGCCATGGAACGTCAGGC-3′; antisense: 5′-CTCCAGAGTTGCGGTTGTGG-3′. *Wnt5a*-sense: 5′-AGACCTCAGAGGGGATGGAC-3′; antisense: 5′-TCTCCGTGCACTTCTTGCAT-3′. *18S*-sense: 5′-TAACCCGTTGAACCCCATT-3; antisense: 5′-CCATCCAATCGGTAGTAGCG-3′.

### 4.13. RNA Sequencing Analysis

After extracting total RNA (5 µg) from cerebral cortex samples, mRNA was purified using Dynabeads Oligo(dT) (Thermo Fisher, Waltham, MA, USA), involving two purification steps. The purified mRNA was then cleaved into smaller fragments at 94 °C for 5–7 min. These fragments were reverse-transcribed into cDNA using SuperScript™ II Reverse Transcriptase (Invitrogen, cat. 1896649, Carlsbad, CA, USA). The resulting cDNA served as a template for synthesizing U-labeled second-strand DNAs with *E. coli* DNA polymerase I, RNase H from NEB, and dUTP solution (Thermo Fisher, cat. R0133, Waltham, MA, USA). The ends of these DNA strands were blunted and ligated with dual-index adapters, followed by size selection using AMPure XP beads. The U-labeled second-strand DNAs were treated with a heat-labile UDG enzyme from NEB and then underwent PCR amplification. The PCR protocol was performed as follows: 95 °C for 3 min, followed by eight cycles of denaturation at 98 °C for 15 s, 60 °C for 15 s, and 72 °C for 5 min. The final cDNA libraries had an average insert size of 300 ± 50 bp. Finally, the Illumina NovaSeq™ 6000 system (Illumina, San Diego, CA, USA) was utilized to perform 2 × 150 bp paired-end sequencing (PE150), following the manufacturer’s protocol. This analysis was conducted by LC-Bio Technology Co., Ltd. in Hangzhou, China.

### 4.14. Biostatistical Analysis

The statistical analysis was carried out by using GraphPad Prism biostatistics (GraphPad Prism 9.0, San Diego, CA, USA). The significant differences between control and treatment groups were analyzed by one-way ANOVA or unpaired *t*-test. All the data were expressed by mean ± SEM, and *p* < 0.05 indicated statistical significance.

## Data Availability

The data presented in this study are available upon request from the corresponding author.

## Supplementary Materials

The following supporting information can be downloaded at https://www.mdpi.com/article/10.3390/ijms26062804/s1.
