# Supplementary material for: TBG096 Ameliorates Memory Deficiency in AD Mouse Model via Promoting Neurogenesis and Regulation of Hsc70/HK2/PKM2/LAMP2A Signaling Pathway
Source: Int J Mol Sci. 2025 Mar 20;26(6):2804. doi: 10.3390/ijms26062804 (PMC11943016; doi:10.3390/ijms26062804)
Supplement: Supplementary file 1 [file ijms-26-02804-s001.zip › ijms-3475170-supplementary.pdf]

Supplementary Information

TBG096 Ameliorates Memory Deficiency in AD Mouse Model via Promoting Neurogenesis and Regulation of Hsc70/HK2/PKM2/LAMP2A Signaling Pathway

Danni Chen[1], Opeyemi B. Fasina[1], Jiahui Lin[1], Jiayuan Zeng[1], Majid Manzoor[1], Hiroshi Ohno[2], Lan Xiang[1*] and Jianhua Qi[1*]

[1]College of Pharmaceutical Sciences, Zhejiang University, Yu Hang Tang Road 866, Hangzhou 310058, China

[2]RIKEN Center for Integrative Medical Sciences, 1-7-22 Suehirocho, Tsutumiku, Yokohama, Japan

[*]Correspondence should be addressed to Lan Xiang, lxiang@zju.edu.cn; Jianhua Qi, qijianhua@zju.edu.cn

1. Chemistry

*1.1 Synthesis and Purification of Compounds **1–4***

The synthesis route of compounds **1–4** is as follows:

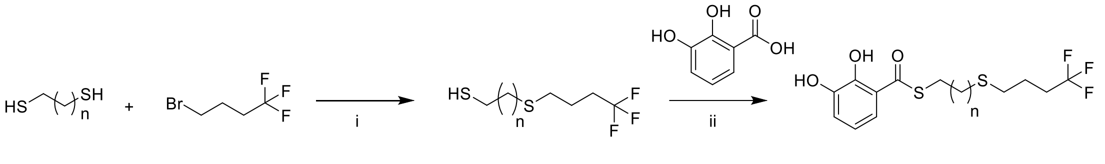

1–4

n=1, 3, 5, 7

Figure S1. The synthesis route of compounds **1–4**. Reagents and conditions: (i).NaH, TBAI, DMF, 0°C to rt, overnight; (ii). EDC·HCl, DMAP, DCM, rt, 24 h.

The synthesis and purification methods of compounds **1, 2, and 4** are as follows:

The synthesis and purification method of compounds **1:** 1,2-Ethanedithiol (188.0 mg, 2.0 mmol) was dissolved in anhydrous N, N-dimethylformamide (DMF) (10 mL). 60% NaH (180.0 mg, 4.5 mmol) was added in at 0°C, kept stirring for 30 min, and warmed up to room temperature for another 30 min. 1-bromo-4,4,4-trifluorobutane (0.3 mL, 2.0 mmol) and tetrabutylammonium iodide (TBAI) (74.0 mg, 0.2 mmol) were added at 0°C. The mixture was stirred overnight at room temperature. The reaction was stopped by adding 50 mL of EtOAc, the mixture was washed with 1 N HCl solution, water, and saturated aqueous solution of $NaHCO_3$ and NaCl, successively. The organic phase was dried over Na_2SO_4, filtered, and then concentrated. The product of the first step was dissolved in anhydrous CH_2Cl_2 (10 mL), DMAP (244 mg, 2.0 mmol), EDC·HCl (382.0 mg, 2.0 mmol), and 2,3-dihydroxybenzoic acid (154.0 mg, 1.0 mmol) was added into this mixture. The mixture was stirred overnight at room temperature, and then concentrated under vacuum. The crude mixture was purified by silica gel open column (*n*-hexane: EtOAc=80 : 1) and ODS open column (Methanol: H_2O=75 : 25) to afford compound **1** (19.7 mg, 5.8%).

Compound 1: HR ESI-TOF-MS *m/z* 363.0316, calcd. for $C_{13}H_{15}F_3O_3S_2Na$ $[M+Na]^+$ 363.0312. ^1H NMR (500 MHz, $CDCl_3$): δ = 11.08 (1H, s), 7.39 (1H, dd, *J*

=1.4, 8.0 Hz), 7.12 (1H, dd, J =1.3, 8.0 Hz), 6.83 (1H, t, J =8.0 Hz), 5.69 (1H, s), 3.27 (2H, m), 2.79 (2H, m), 2.71 (2H, t, J =7.1 Hz), 2.25 (2H, m), 1.93 (2H, m).

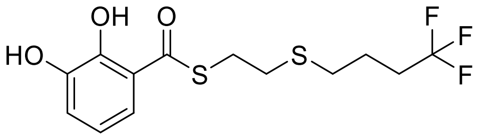

1

The synthesis and purification method of compounds **2:** 1,4-Butanedithiol (244.0 mg, 2.0 mmol) was dissolved in anhydrous N, N-dimethylformamide (DMF) (10 mL). 60% NaH (180 mg, 4.5 mmol) was added in at 0°C, kept stirring for 30 min, and warmed up to room temperature for another 30 min. 1-bromo-4,4,4-trifluorobutane (0.3 mL, 2.0 mmol) and tetrabutylammonium iodide (TBAI) (74.0 mg, 0.20 mmol) were added at 0°C. The mixture was stirred overnight at room temperature. The reaction was stopped by adding 50 mL of EtOAc, the mixture was washed with 1 N HCl solution, water, and saturated aqueous solution of $NaHCO_3$ and NaCl, successively. The organic phase was dried over Na_2SO_4, filtered, and then concentrated. The product of the previous step was dissolved in anhydrous CH_2Cl_2 (10 mL), DMAP (244 mg, 2.0 mmol), EDC·HCl (382.0 mg, 2.0 mmol), and 2,3-dihydroxybenzoic acid (154.0 mg, 1.00 mmol) was added into this mixture. The mixture was stirred overnight at room temperature, and then concentrated under vacuum. The crude mixture was purified by silica gel open column (*n*-hexane: EtOAc=80 : 1) and ODS open column (Methanol: H_2O=75 : 25) to afford compound **2** (35.3 mg, 9.6%).

Compound 2: HR ESI-TOF-MS *m/z* 391.0625, calcd. for $C_{15}H_{19}F_3O_3S_2Na$ $[M+Na]^+$ 391.0625. ^1H NMR (500 MHz, $CDCl_3$): δ = 11.19 (1H, s), 7.41 (1H, dd, J =1.4, 8.0 Hz), 7.11 (1H, dd, J =1.4, 8.0 Hz), 6.82 (1H, t, J =8.0 Hz), 5.69 (1H, s), 3.09 (2H, t, J =7.1 Hz), 2.57 (4H, m), 2.22 (2H, m), 1.83 (4H, m), 1.73 (2H, m).

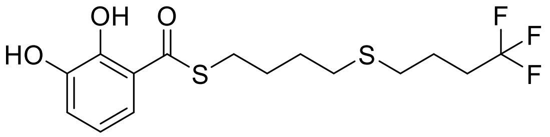

2

The synthesis and purification method of compounds **4:** 1,8-Octanedithiol (357.0 mg, 2.0 mmol) was dissolved in anhydrous N, N-dimethylformamide (DMF) (10 mL). 60% NaH (180.0 mg, 4.5 mmol) was added in at 0°C, kept stirring for 30 min and warmed up to room temperature for another 30 min. 1-bromo-4,4,4-trifluorobutane (0.45 mL, 3.0 mmol) and tetrabutylammonium iodide (TBAI) (74.0 mg, 0.2 mmol) were added in at 0°C. The mixture was stirred overnight at room temperature. The reaction was stopped by adding 50 mL of EtOAc, the mixture was washed with 1 N HCl solution, water, and saturated aqueous solution of $NaHCO_3$ and NaCl, successively. The organic phase was dried over Na_2SO_4, filtered, and then concentrated. The product was dissolved in anhydrous CH_2Cl_2 (10 mL), DMAP (244.0 mg, 2.0 mmol), EDC·HCl (382.0 mg, 2.0 mmol), and 2,3-dihydroxybenzoic acid (154 mg, 1.00 mmol) was added into this mixture. The mixture was stirred overnight at room temperature, and then concentrated under vacuum. The crude mixture was purified by silica gel open column (*n*-hexane: EtOAc=80 : 1) and ODS open column (Methanol: H_2O=85 : 15) to afford compound **4** (20.4 mg, 4.8%).

Compound 4: HR ESI-TOF-MS *m/z* 447.1257, calcd. for $C_{19}H_{27}F_3O_3S_2Na$ $[M+Na]^+$ 447.1251. ^1H NMR (500 MHz, $CDCl_3$): δ = 11.25 (1H, s), 7.41 (1H, dd, *J* =1.4, 8.0 Hz), 7.10 (1H, dd, *J* =1.4, 8.0 Hz), 6.81 (1H, t, *J* =8.0 Hz), 5.68 (1H, s), 3.06 (2H, t, *J* =7.3 Hz), 2.54 (4H, m), 2.22 (2H, m), 1.85 (2H, m), 1.68 (2H, m), 1.60 (2H, m), 1.37 (8H, m).

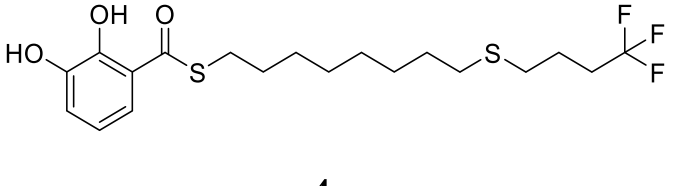

4

*1.2 ^{1}H NMR and ^{13}C NMR Spectrums of Compound **3** (TBG096)*

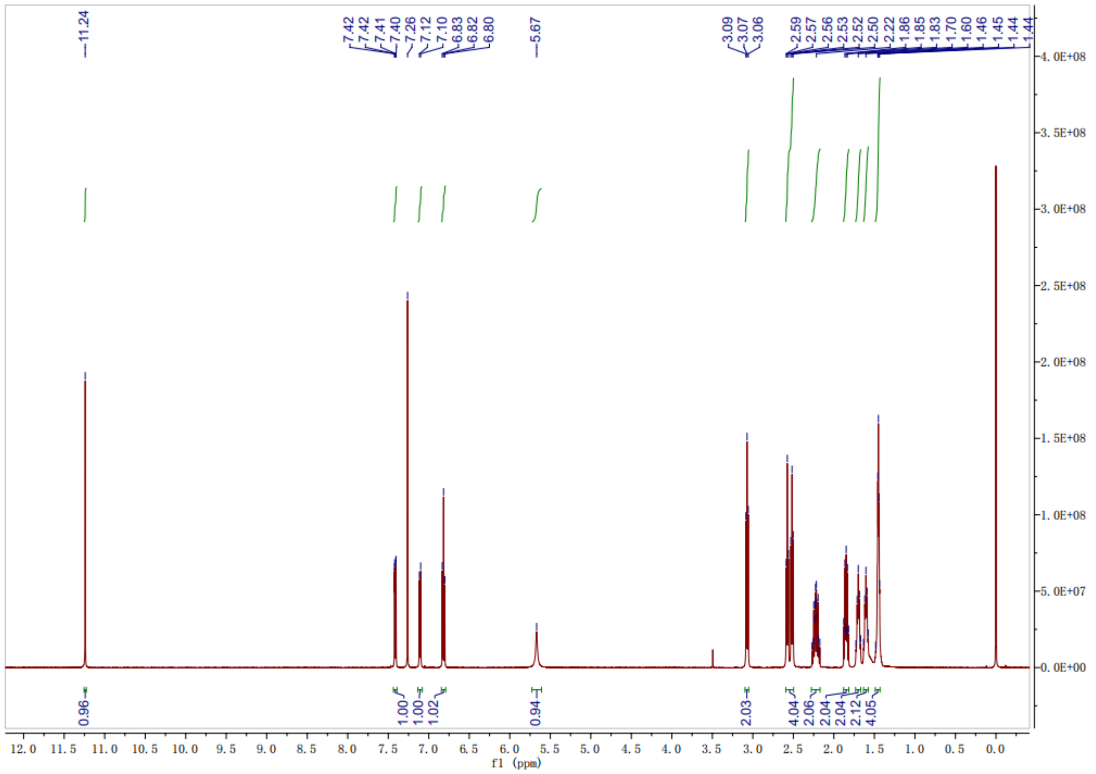

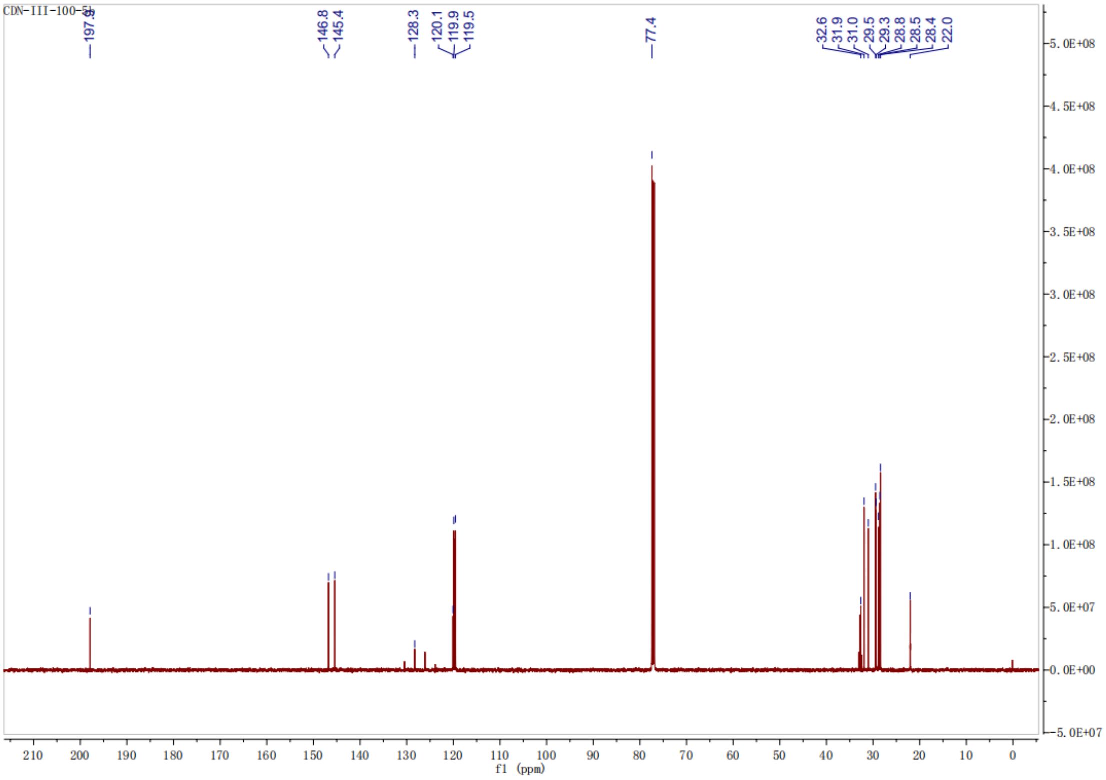

Figure S2. ^{1}H NMR (500 MHz, $CDCl_3$) and ^{13}C NMR (125 MHz, $CDCl_3$) spectrums of TBG096

2. Biology

2.1 Related Indexes of Acute Toxicity Test in Mice

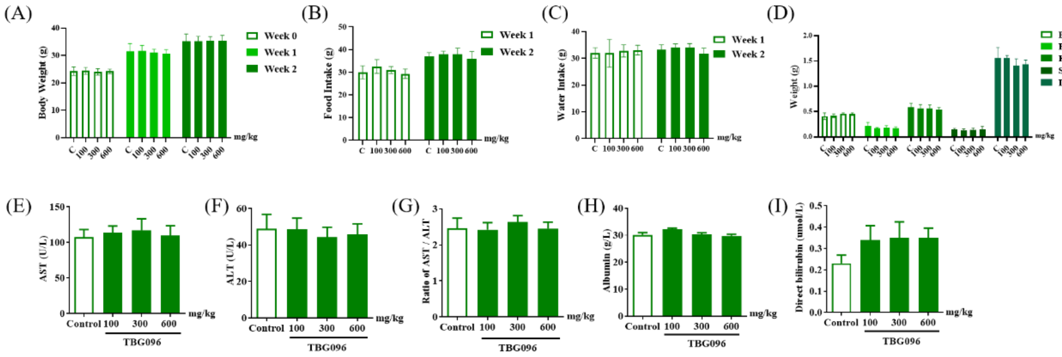

Figure S3. Related indexes of acute toxicity test in mice after oral administration of TBG096 at 100, 300, and 600 mg/kg. Changes in **(A)** body weight, **(B)** food intake, **(C)** water intake, **(D)** organ weight, and **(E-I)** serum AST, ALT, AST/ALT, ALB, and DBIL levels of ICR mice after administering the TBG096 at doses of 100, 300, and 600 mg/kg for two weeks.

2.2 *The HPLC and HR ESI-TOF-MS Analysis of Plasma Samples after Administration of TBG096*

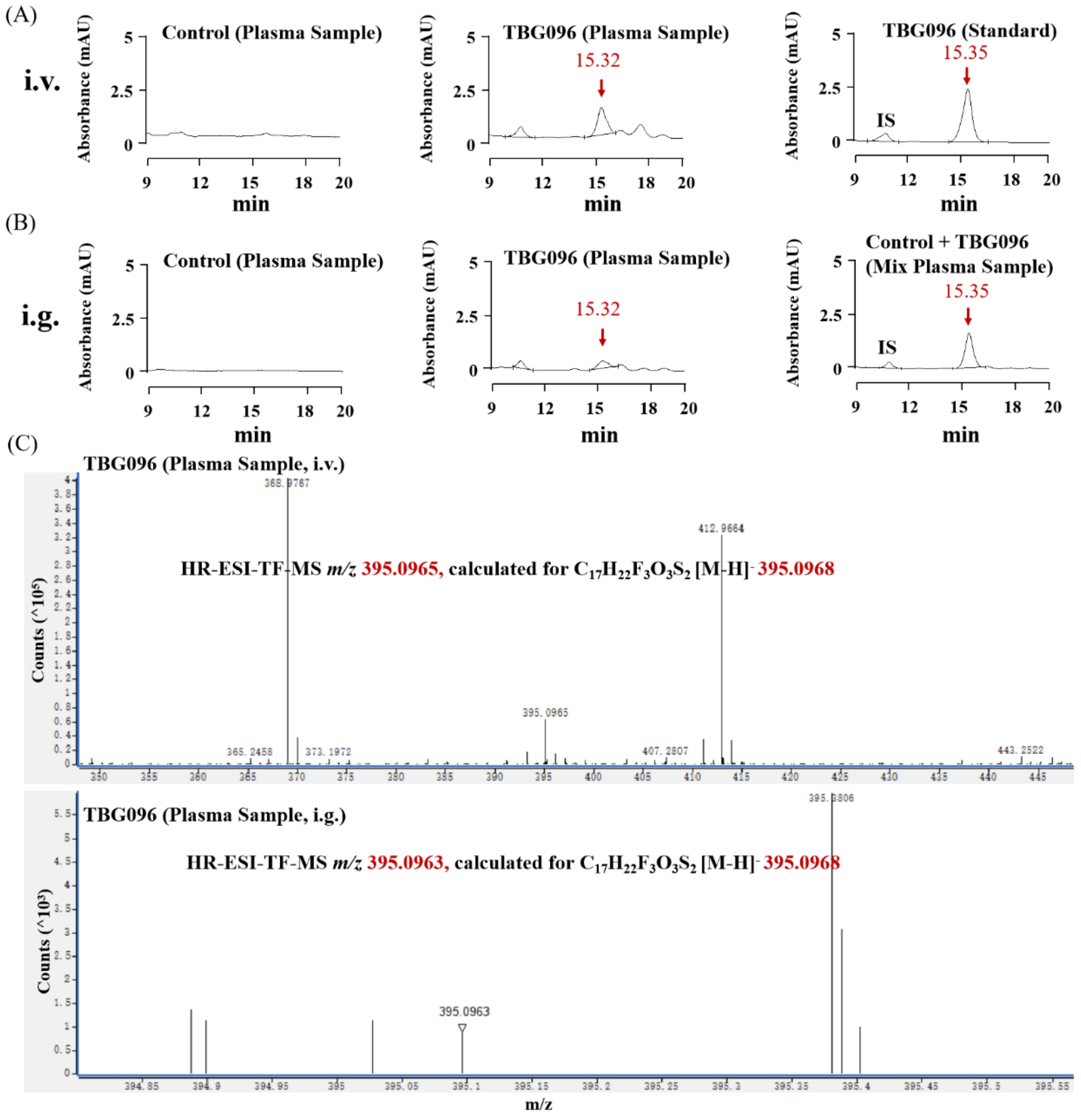

Figure S4. HPLC and HR ESI-TOF-MS analysis of the plasma after TBG096 administration in rats. **(A)** HPLC analysis results of plasma samples in control, TBG096 group (14 mg/kg) after 5 minutes of intravenous administration, TBG096 standard and internal standard (IS, compound **2**). **(B)** HPLC analysis results of plasma samples in control, TBG096 group (14 mg/kg) after 15 min of oral administration, mixed control sample (including TBG096 and internal standard (IS, compound **2**)). **(C)** High-resolution mass spectrometry of collected HPLC peak from plasma samples after a single intravenous or oral administration of TBG096 (14 mg/kg).

2.3 The Sequencing Analysis of Transcriptome in the Cerebral Cortex of HFD-Induced AD Mice

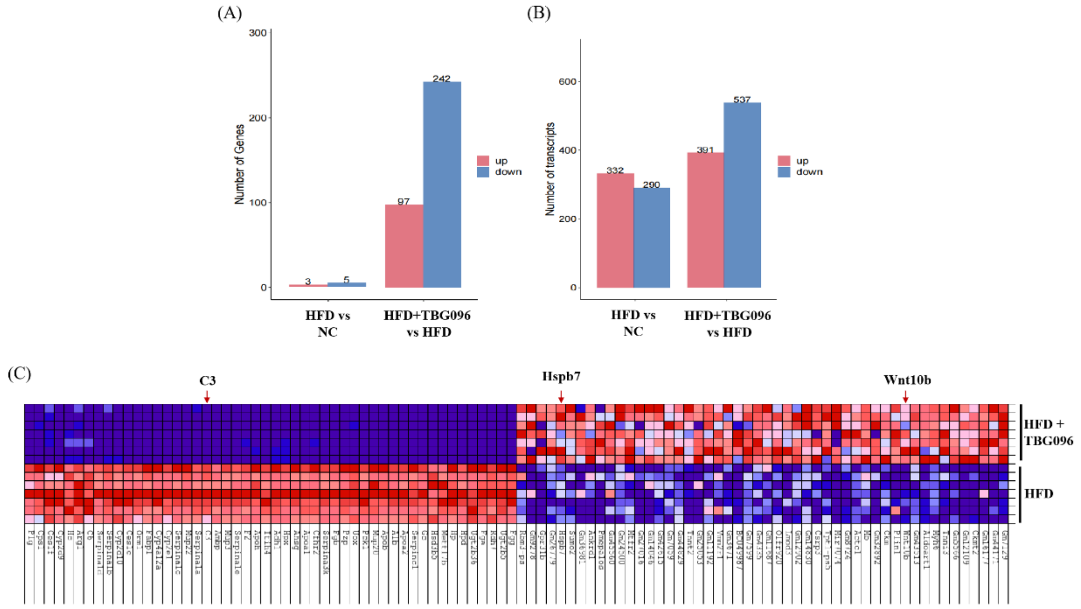

Figure S5. The sequencing analysis of transcriptome in the cerebral cortex of HFD-induced AD mice. **(A)** and **(B)** the changed number of genes and transcripts. **(C)** Heat map of top 100 changed genes in HFD+TBG096 group compared with HFD group. The red color represents up-regulation and the blue color represents down-regulation.

2.4 Inhibitor Experiment Result of NGF-Mimic Activity

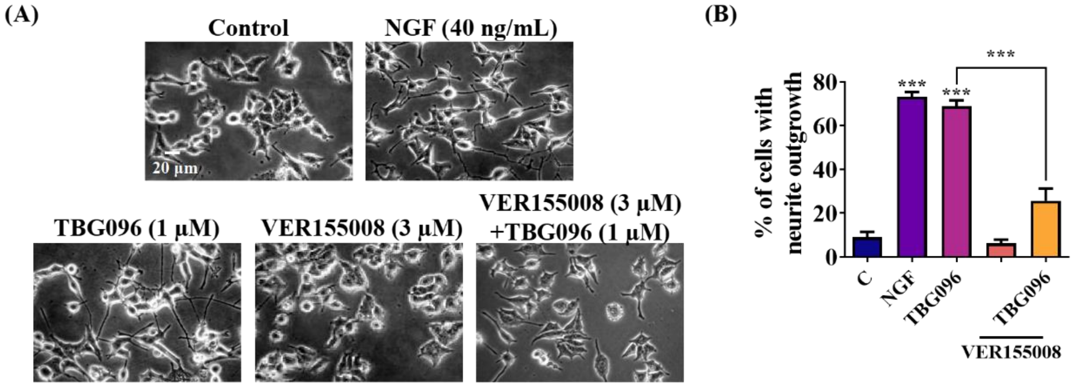

Figure S6. Effects of VER155008 (inhibitor of Hsc70) on the NGF-mimic activity of TBG096 in PC12 cells. **(A)** Morphological changes and **(B)** digital results of the inhibitor of Hsc70 (VER155008) on the NGF-mimic effect of TBG096 in PC12 cells.

2.5 Origin Data of Western Blot Analysis for Signaling Pathways of TBG096

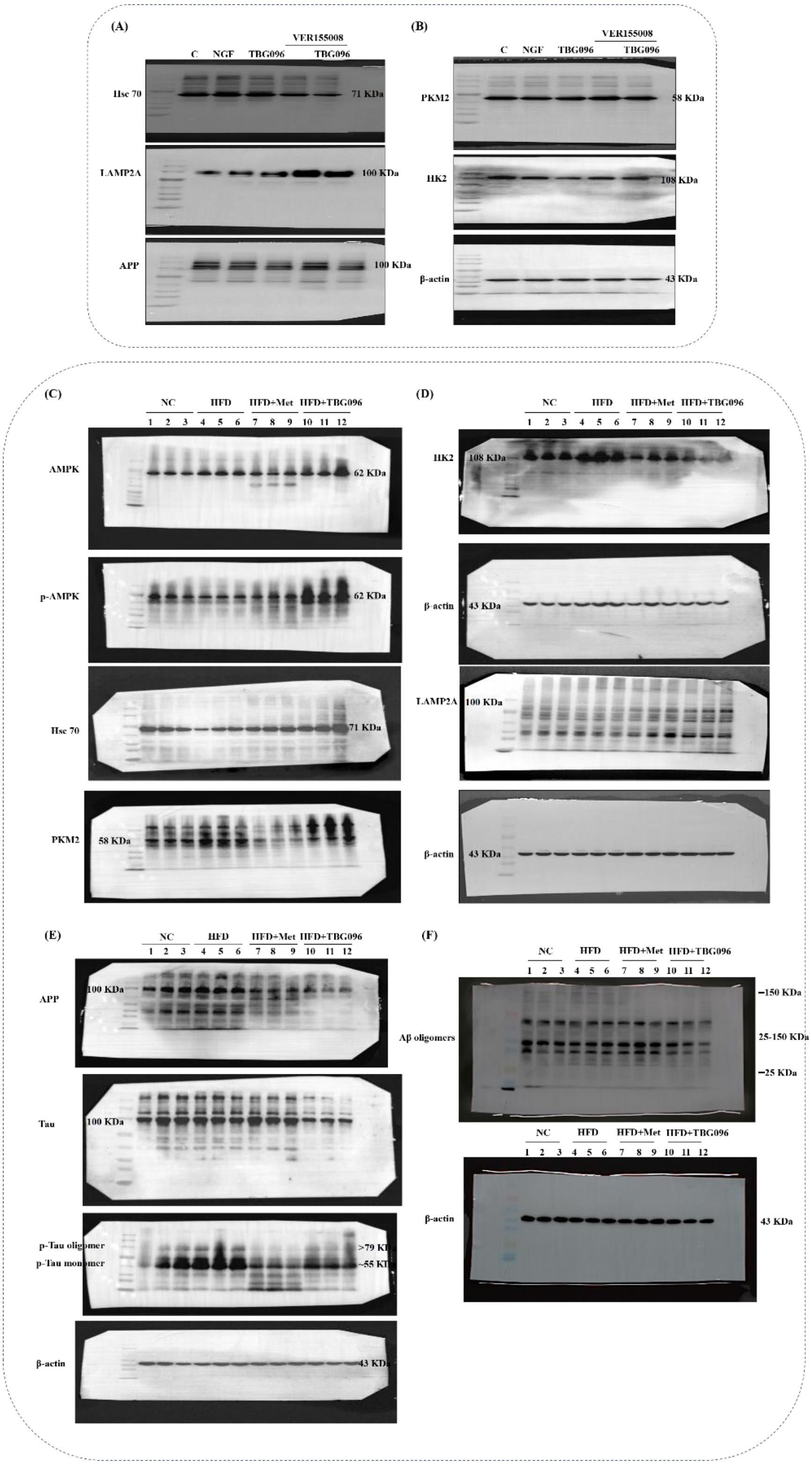

Figure S7. The original data of western blot analysis in **Figure 5A, 5J,** and **5L**. **(A-B)** Western blot analysis of Hsc70, LAMP2A, APP, PKM2, and HK2 proteins in PC12 cells. **(C-D)** Western blot

analysis of AMPK, p-AMPK, Hsc70, PKM2, HK2, and LAMP2A proteins in the cerebral cortex of HFD-induced AD mice. **(E-F)** Western blot analysis of APP, Tau, p-Tau, and Aβ oligomers proteins in the cerebral cortex of HFD-induced AD mice.

2.6 Behavior Test Results of HFD-Induced AD Mice

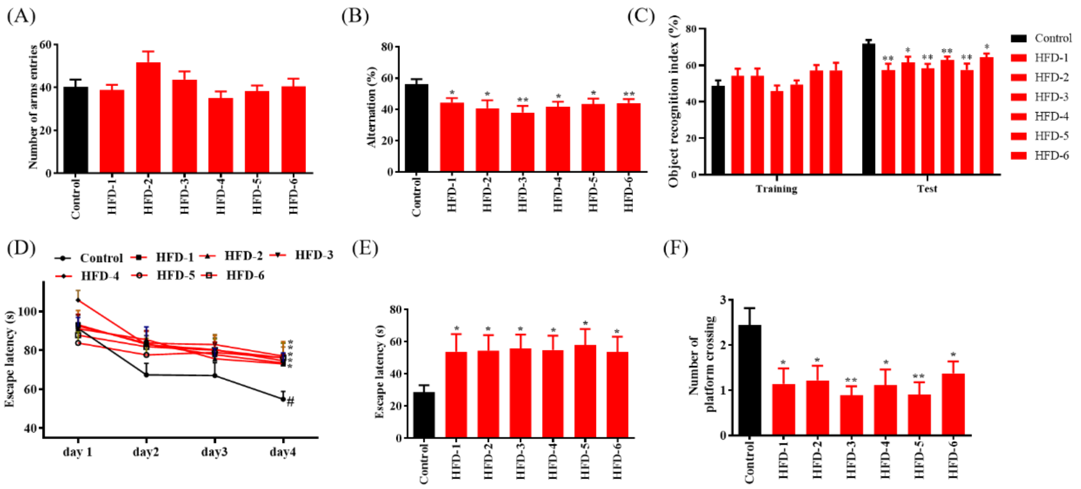

Figure S8. Changes in learning memory and spatial memory of AD mice. HFD-induced AD mice: **(A)** Changes in the number of arm entries in the Y-maze test. **(B)** Percentage of alternation in the Y-maze test. **(C)** Percentage of object recognition index in the NOR test. **(D)** Escape latency in the training phase of the MWM test. **(E)** Escape latency in the test phase of the MWM test. **(F)** Platform crossing numbers in the test phase of the MWM test.

2.7 TBG096 Remodels Gut Microbiota in AD Mice Therapeutically

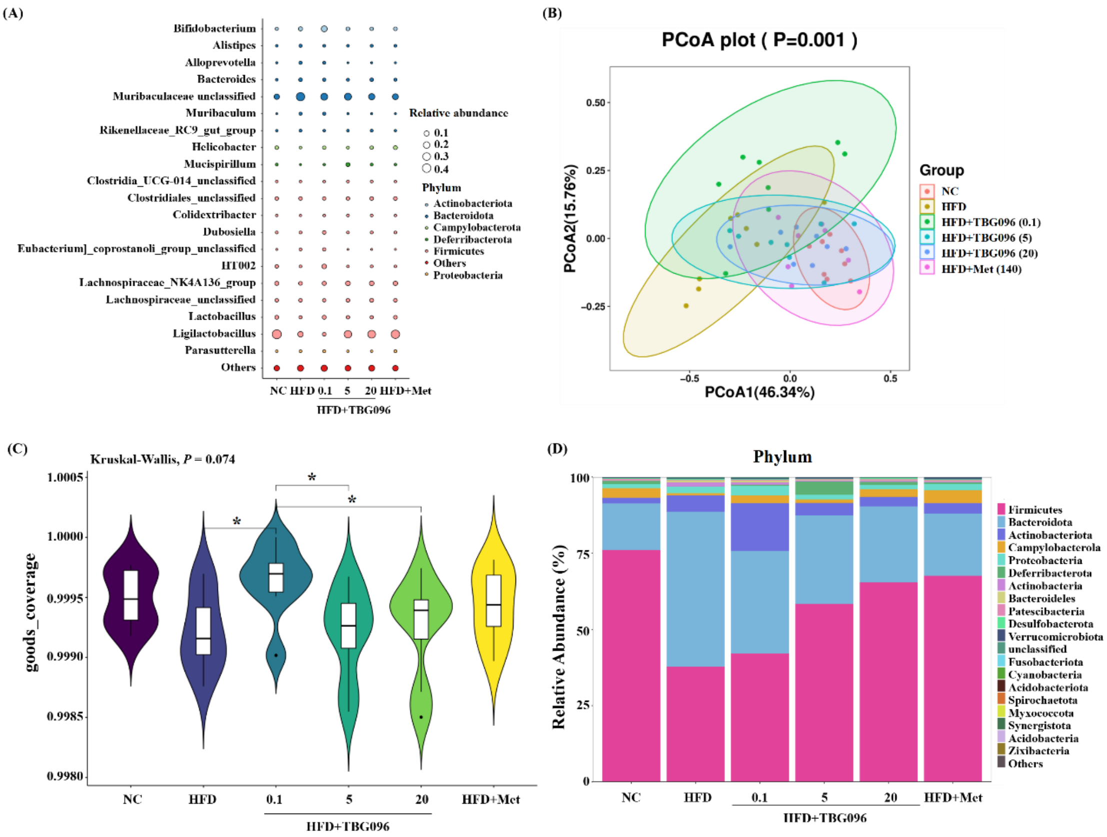

Figure S9. TBG096 changes the diversity and composition of gut microbiota in AD mice. **(A)** The bubble plot of taxonomy analysis in the phylum of gut microbiota in each group (NC: control group). **(B)** The PCoA plot of each group. **(C)** The violin plot of α diversity analysis in each group. **(D)** Relative abundance (%) analysis in the phylum of each group. The sample number of each group is eight. * indicates a significant difference at $q < 0.05$.
